# Epigenetic integrity of paternal imprints enhances the developmental potential of androgenetic haploid embryonic stem cells

**DOI:** 10.1007/s13238-021-00890-3

**Published:** 2021-12-05

**Authors:** Hongling Zhang, Yuanyuan Li, Yongjian Ma, Chongping Lai, Qian Yu, Guangyong Shi, Jinsong Li

**Affiliations:** 1 State Key Laboratory of Cell Biology, Shanghai Key Laboratory of Molecular Andrology, CAS Center for Excellence in Molecular Cell Science, Shanghai Institute of Biochemistry and Cell Biology, University of Chinese Academy of Sciences, Chinese Academy of Sciences, Shanghai, 200031 China; 2 School of Life Science and Technology, ShanghaiTech University, Shanghai, 201210 China; 3 Animal Core Facility, Shanghai Institute of Biochemistry and Cell Biology, Center for Excellence in Molecular Cell Science, Chinese Academy of Sciences, University of Chinese Academy of Sciences, Shanghai, 200031 China; 4 School of Life Science, Hangzhou Institute for Advanced Study, University of Chinese Academy of Sciences, Hangzhou, 310024 China

**Keywords:** paternal imprints, androgenetic haploid ESCs, DMRs, semi-cloned mice, alternative 2i

## Abstract

The use of two inhibitors of Mek1/2 and Gsk3β (2i) promotes the generation of mouse diploid and haploid embryonic stem cells (ESCs) from the inner cell mass of biparental and uniparental blastocysts, respectively. However, a system enabling long-term maintenance of imprints in ESCs has proven challenging. Here, we report that the use of a two-step a2i (alternative two inhibitors of Src and Gsk3β, TSa2i) derivation/culture protocol results in the establishment of androgenetic haploid ESCs (AG-haESCs) with stable DNA methylation at paternal DMRs (differentially DNA methylated regions) up to passage 60 that can efficiently support generating mice upon oocyte injection. We also show coexistence of H3K9me3 marks and ZFP57 bindings with intact DMR methylations. Furthermore, we demonstrate that TSa2i-treated AG-haESCs are a heterogeneous cell population regarding paternal DMR methylation. Strikingly, AG-haESCs with late passages display increased paternal-DMR methylations and improved developmental potential compared to early-passage cells, in part through the enhanced proliferation of *H19*-DMR hypermethylated cells. Together, we establish AG-haESCs that can long-term maintain paternal imprints.

**Supplementary Information:**

The online version contains supplementary material available at 10.1007/s13238-021-00890-3.

## INTRODUCTION

Culture conditions with 2i (inhibitors of Mek1/2 and Gsk3β) supplemented with leukemia inhibitory factor (LIF) (2i/L) enhance the derivation of embryonic stem cells (ESCs) with naïve ground-state pluripotency (Ying et al., 2008). However, recent studies indicate that prolonged application of 2i results in a widespread loss of DNA methylation, including repetitive elements and imprinted genes, and impaired developmental potential in ESCs (Choi et al., 2017b; Yagi et al., 2017). An alternative 2i/L (Mek1/2 inhibitor is substituted with the Src inhibitor, known as a2i/L) has been developed (Shimizu et al., 2012) and used to preserve the epigenetic integrity and developmental potential of ESCs (Choi et al., 2017b; Yagi et al., 2017). Nevertheless, the long-term culture of female ESCs in a2i/L induced a reduction of ICR (imprinting control region) methylation (Yagi et al., 2017). Meanwhile, although male ESCs under a2i/L can stable maintain DMR methylation until passage 15 (Yagi et al., 2017), whether prolonged culture ensures intact DMR methylation is still unknown. Similarly, the conventional serum plus LIF (S/L) medium also induced loss of DNA methylation at ICRs in mouse ESCs upon prolonged culturing (Dean et al., 1998; Humpherys et al., 2001; Yagi et al., 2017). Therefore, there is an unmet need for the establishment or employment of appropriate derivation and culture conditions that would enable the long-term stable maintenance of imprinting marks in ESCs.

Application of 2i/L culture conditions has enabled the successes in deriving mouse haploid ESCs (haESCs) from parthenogenetic and androgenetic embryos (termed PG-haESCs and AG-haESCs, respectively) (Elling et al., 2011; Leeb and Wutz, 2011; Li et al., 2012; Yang et al., 2012). Both PG and AG-haESCs can be employed for high-throughput genetic analyses at a cellular level (Elling et al., 2011; Leeb et al., 2014; Leeb and Wutz, 2011; Sagi et al., 2016; Yang et al., 2013; Zhong et al., 2016b). Notably, AG-haESCs can be used as sperm replacement to support embryonic development upon injection into oocytes, leading to the production of semi-cloned (SC) embryos (Li et al., 2012; Yang et al., 2012; Zhang et al., 2020). However, 2i/L-treated PG-haESCs were globally hypomethylated (Choi et al., 2017a; Elling et al., 2011; Zhong et al., 2016a). Meanwhile, AG-haESCs lost DNA methylation at differentially DNA methylated regions (DMRs), i.e., *H19*-DMR and *IG*-DMR, that control two paternal imprinted loci, *H19*-*Igf2* and *Dlk1*-*Dio3*, respectively, resulting in the low birth rate of SC mice (Yang et al., 2012). Interestingly, deleting both DMRs in AG-haESCs (termed DKO-AG-haESCs) could decrease the expression of *H19* and *Gtl2* and dramatically rescue the developmental potential of resultant SC embryos (Li et al., 2020; Zhong et al., 2015). We reasoned that, if AG-haESCs could be generated under a2i/L conditions, they might stably maintain paternal imprinting features at least at early passages, which allows for the better developmental potential of SC embryos without removal of paternal DMRs and thus provides a useful tool for an efficient combination of *in vitro* and *in vivo* studies of imprinting regulation.

In this study, we tested our hypothesis using a2i/L with serum, w/o serum or a two-step protocol (Yagi et al., 2017) (a2i/L with serum for derivation followed by a2i/L without serum for maintenance, TSa2i/L) and found that TSa2i/L enabled generating AG-haESCs with stable DNA methylation in paternal DMRs up to 60 passages. Furthermore, we showed that H3K9me3 deposition and ZFP57 binding are preserved at *H19* and *IG* DMRs and highly correlated with DMR methylation, which are likely involved in the stable maintenance of paternal imprints in TSa2i/L-derived AG-haESCs. In contrast, H3K4me3 deposition may prevent DMRs from *de novo* methylation upon loss of imprints. Strikingly, we found that TSa2i/L-treated AG-haESCs with late passages exhibited higher paternal DMR methylations and increased developmental potential compared to cells of early passages. We further demonstrated that TSa2i/L-treated AG-haESCs are a heterogeneous cell population regarding *H19*-DMR methylation levels and cells with hypermethylated *H19*-DMR display better proliferation potential compared to cells with hypomethylated *H19*-DMR, partially accounting for long-term maintenance of paternal imprints.

## RESULTS

### A two-step a2i/L derivation/culture condition enables generation of AG-haESCs with hypermethylated paternal DMRs

To derive haploid ESCs, we reconstructed haploid embryos through sperm nuclear transfer, i.e., injection of sperm head into enucleated oocytes (Yang et al., 2012). The sperm-cloned embryos developed to the blastocyst stage *in vitro*, followed by ESC derivation in one of three culture conditions, S/L, S/L supplemented with 2i (2i/L) or S/L with a2i (a2i/L). Both 2i/L and a2i/L conditions promoted the derivation of ESCs from sperm-cloned blastocysts compared to S/L (Fig. S1A and S1B). Among 12 2i/L-derived and 8 a2i/L-derived ESC lines, 7 and 4 of them respectively contained a subpopulation of haploid cells that can be enriched and maintained through regular fluorescence-activated cell sorting (FACS) (Fig. S1B–D). Consistent with our previous observations (Yang et al., 2012), 2i/L-derived AG-haESCs could give rise to SC mice upon intracytoplasmic AG-haESC injection (ICAHCI) at low efficiency at early passages, but lost the ability upon prolonged culturing, due to decreased DNA methylation at *H19*-DMR and *IG*-DMR (Li et al., 2020; Zhong et al., 2015) (Fig. S1E and S1F; Table S1). Expectedly, a2i/L-cultured haploid cells of early passages sustained DNA methylation at *H19*-DMR and *IG*-DMR and exhibited better developmental potential demonstrated by ICAHCI compared to 2i/L cells (Fig. S1G and S1H; Table S1). However, DNA methylation of paternal DMRs was progressively reduced in a2i/L-cultured haploid cells of later passages, leading to the decreased birth rate of SC mice (Fig. S1H; Table S1), most likely due to the use of serum that causes loss of DNA methylation at DMRs in diploid ESCs upon prolonged culturing (Dean et al., 1998; Humpherys et al., 2001; Yagi et al., 2017).

**Figure 1:**
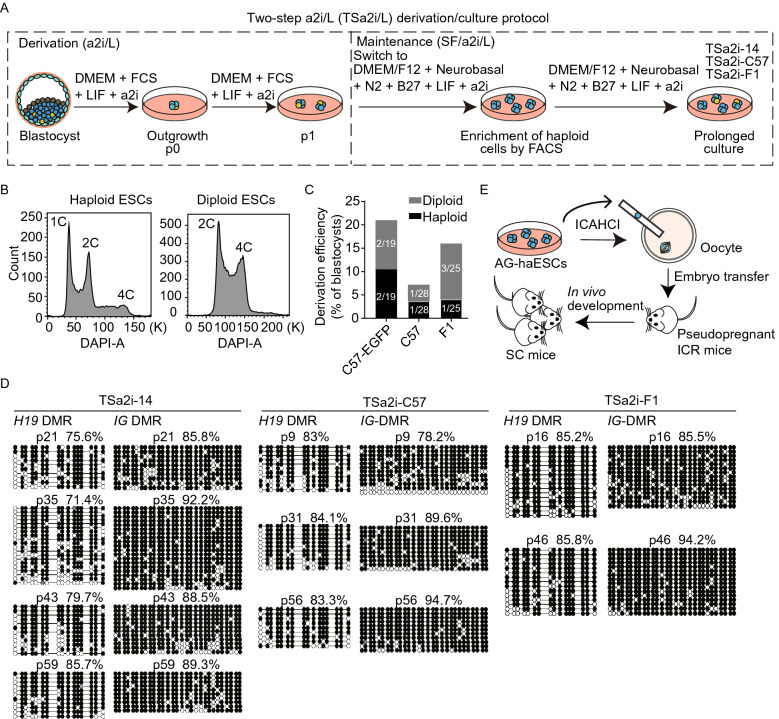
**Derivation of ground-state AG-haESCs maintaining hypermethylated paternal DMRs**. (A) Diagram for the two-step derivation/culture protocol of AG-haESCs in a2i/L medium (TSa2i/L protocol). TSa2i-14, TSa2i-C57, and TSa2i-F1 are cell lines derived using TSa2i/L protocol, which were studied in this study. Haploid and diploid cells were labeled in blue and yellow, respectively. Haploid cells were enriched by FACS. (B) Establishment of AG-haESCs by FACS enrichment for haploid cells (Left). A DAPI filter was used to detect a signal of Hoechst 33342-stained DNA. Right, FACS data of diploid ESCs for comparison. (C) Summary of diploid and haploid ESC lines derived using TSa2i/L method from cloned blastocysts generated from sperm with different genetic backgrounds. Diploid cells are from diploidized haploid ESCs during ESC derivation and maintenance. (D) DNA methylation state of *H19* and *IG* DMRs in TSa2i-derived AG-haESCs (TSa2i-14, TSa2i-C57, and TSa2i-F1) with different passages determined by bisulfite sequencing. Open and filled circles represent unmethylated and methylated CpG sites, respectively. (E) Diagram showing the generation of semi-cloned (SC) mice through intracytoplasmic AG-haESC injection (ICAHCI)

We next adopted a well-defined serum-free ESC medium (DMEM/F12 + Nerobasal + N2 + B27 + LIF) supplemented with a2i (termed SF/a2i/L) for AG-haESC derivation. From a total of 25 sperm-cloned blastocysts, we obtained 16 outgrowths. However, none of them could be passaged, indicating that serum or its replacement is critical for ESC derivation (Martello and Smith, 2014). We next employed a two-step protocol (Yagi et al., 2017), in which, a2i/L medium was employed for formation of outgrowths and a few times of cell passaging before the use of SF/a2i/L conditions for long-term maintenance (termed TSa2i/L protocol) (Fig. 1A). We found that one time of expansion in a2i/L followed by transferring into SF/a2i/L could give rise to stable haploid cell lines from different genetic backgrounds through regular FACS-enrichment of haploid cells (Figs. 1B, 1C, S2A, and S2B). Surprisingly, DNA methylations at both *H19* and *IG*-DMRs were stably maintained at high levels upon prolonged culturing (up to passage 59, p59) in three tested lines (TSa2i-14, TSa2i-C57, and TSa2i-F1), although *Rasgrf1*-DMR was not stable in these cell lines (Figs. 1D and S2C). Consistently, ICAHCI analysis showed that all three tested TSa2i/L-treated haploid cells at early passages (<p30) efficiently supported the full-term embryonic development of SC embryos (Fig. 1E; Table 1), reaching an efficiency similar to DKO-AG-haESCs cultured in 2i/L (Zhong et al., 2015). Meanwhile, while cesarean section (C-section) was required to obtain SC pups when 2i/L or a2i/L-cultured cells were used for ICAHCI, the recipient females delivered TSa2i/L-treated-cell-derived SC pups by themselves similar to those carrying SC embryos derived from DKO-AG-haESCs (Fig. S2D) (Zhong et al., 2015).

**Table 1:** Summary of SC mice derived from TSa2i/L-derived AG-haESCs

AG-haESC line	Genetic background/gene-modifications	Passage number	No. of transferred 2-cell embryos	No. of growth-retarded pups (% transferred embryos)^a^	No. of normal pups (% transferred embryos)^a^
TSa2i-14	C57BL/6/actin-EGFP	p23-p28	252	ND	36 (14.3)
		p33-p54	460	ND	107 (23.3)
		p60	144	2 (1.4)^a^	45 (31.3)^a^
TSa2i-C57	C57BL/6	p12-p24	252	ND	43 (17.1)
		p21	114	1 (0.9)^a^	17 (14.9)^a^
		p30-p62	507	ND	141 (27.8)
TSa2i-F1	B6D2F1	p12-p27	404	ND	87 (21.5)
		p35	190	0^a^	46 (24.2)^a^
	Subtotal	p12-p30	1,022		183 (17.9)
		p30-p62	1,301		339 (26.1)
TSa2i-14-Tet3 KO	C57BL/6/actin-EGFP /*Tet3* KO	p38	144	ND	38 (26.4)
TSa2i-14-Tet TKO	C57BL/6/actin-EGFP/*Tet1*, *2*, *3* knockout	p45-p47	243	ND	77 (31.7)
TSa2i-14-*Dusp9*-HA-138	C57BL/6/actin-EGFP/*Dusp9*-HA knockin	p37	114	ND	37 (32.5)
TSa2i-14-*Dusp9*-HA-120	C57BL/6/actin-EGFP/*Dusp9*-HA knockin	p37	144	ND	12 (8.3)
TSa2i-14-H19KO (H19KO-40)	C57BL/6/actin-EGFP/H19-DMR knockout	p68 & p69	360	0^a^	90 (25)^a^
TSa2i-14-DKO (DKO-20)	C57BL/6/actin-EGFP/*H19*-DMR & *IG*-DMR knockout	p68	180	0^a^	43 (23.9)^a^
TSa2i-C57-DKO (DKO-187 p68)	C57BL/6/*H19*-DMR & *IG*-DMR knockout	p68	144	3 (2.1)^a^	32 (22.2)^a^

^a^Derived by C-section at E18.5

ND: not detected

### Prolonged SF/a2i/L culture enhances the developmental potential of AG-haESCs

**Figure 2:**
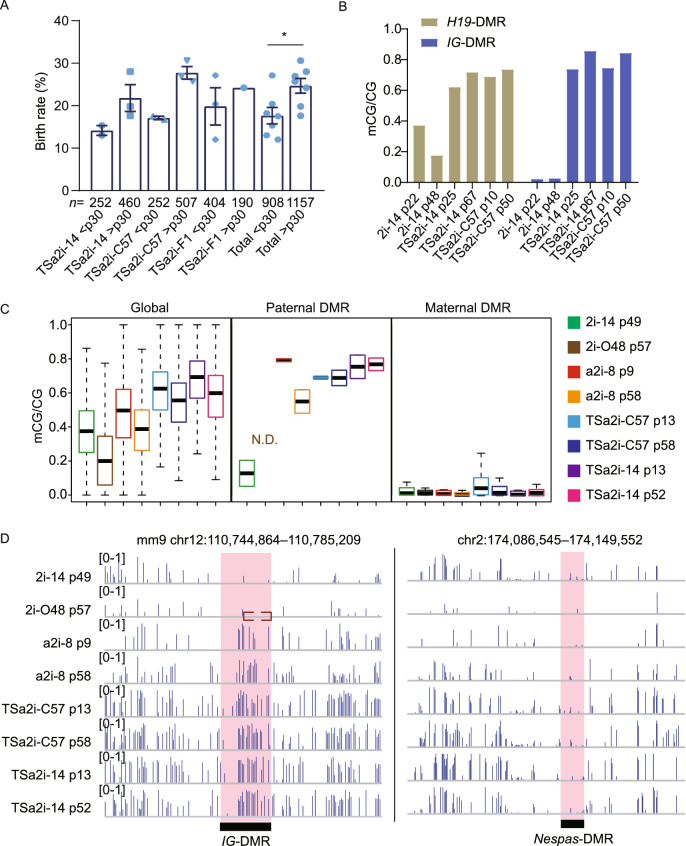
**Prolonged SF/a2i/L culture increases the developmental potential of AG-haESCs by ICAHCI**. (A) Birth rate of the SC mice generated from TSa2i/L-derived cells with early and late passages. Passage 30 is a cutoff used to divide cells into early and late groups. SC pups obtained by cesarean section (C-section) were not used to calculate the birth rate. All error bars indicate the average mean ± SEM. *P* value was calculated by *t* test. *, *P* < 0.05. (B) DNA methylation levels at *H19* and *IG* DMRs of early- and late-passage AG-haESCs derived by TSa2i/L conditions. 2i/L (2i-14) AG-haESCs are as controls. (C) Boxplot showing the DNA methylation levels of genome and gamete DMRs in 2i/L (2i-14 and 2i-O48), a2i/L (a2i-8), and TSa2i/L-derived (TSa2i-C57 and TSa2i-14) AG-ESCs. O48 is a cell line with both *H19-* and *IG*-DMR deletions derived previously (Zhong et al., 2015). N.D., not detected. (D) Snapshots of DNA methylation levels of a paternal DMR (*IG*-DMR) and a maternal DMR (*Nespas*-DMR) in 2i/L (2i-14 and 2i-O48), a2i/L (a2i-8), and TSa2i/L-derived (TSa2i-C57 and TSa2i-14) AG-ESCs. The red dashed box represents the deleted region in 2i-O48

We next examined whether genetic alterations can be introduced into these TSa2i/L-treated AG-haESCs, followed by ICAHCI to efficiently produce SC mice carrying expected genetic traits. To this end, we generated AG-haESCs carrying different genetic modifications, including a single mutant gene (*Tet3*), triple mutant genes (*Tet1*, *2*, and *3*), or a tagged gene (*Dusp9*-HA) (Choi et al., 2017a) by performing CRISPR-Cas9-based genetic manipulations (Fig. S2E and S2F). Surprisingly, all gene-modified TSa2i/L-treated haploid cells could give rise to corresponding SC mice via ICAHCI at a higher efficiency compared to haploid cells before manipulation (Table 1). Interestingly, wildtype TSa2i/L-cultured haploid cells, upon prolonged *in vitro* culture (p30-p62), also exhibited better ability to produce SC mice via ICAHCI (Fig. 2A), thus excluding the possibility that genetic manipulations result in higher developmental potential in haploid cells and suggesting that prolonged period of culturing in SF/a2i/L *per se* may enhance the development of SC embryos. Postnatal growth profiling analysis showed that SC pups obtained from TSa2i/L-treated haploid cells displayed better growth compared to DKO-AG-haESCs (Fig. S2G), probably due to decreased *H19* and increased *Igf2* expression in organs like liver during development (Fig. S2H–K), consistent with the recent observations in 2i/L-cultured AG-haESCs with *H19*, *H19*-DMR and *IG*-DMR deletions (Li et al., 2020).

### Epigenetic integrity of paternal DMRs is preserved in TSa2i/L-cultured AG-haESCs

Given the strong correlation of DNA methylation patterns and *in vivo* developmental potentials of ESCs (Choi et al., 2017b; Yagi et al., 2017; Zwaka, 2017) and previous studies have highlighted that *H19* and *IG* DMR methylation state in AG-haESCs are related to their developmental potential through ICAHCI (Zhang et al., 2015; Zhong et al., 2015), we thus investigated DNA methylation state in cells with prolonged culture. Bisulfite-PCR analysis indicated increased DNA methylation at both of *H19* and *IG* DMRs in TSa2i/L-treated haploid cells of late passages compared to early passages (Fig. 2B). To further assess their methylation patterns, we performed whole-genome bisulfite sequencing (WGBS) using haploid cells of early (<p30) and late passages (p30~p60). The results indicated that TSa2i/L-treated haploid cells sustained high levels of DNA methylation at DMRs of paternally imprinted genes at both early and late passages, even though the genome-wide DNA methylation level showed a slight decrease upon prolonged culture (Figs. 2C and S3A). In contrast, majority of tested canonical maternal DMRs (*n* = 20) were unmethylated in TSa2i/L-treated AG-haESCs during long-term culturing (Figs. 2C, 2D and S3B). We further analyzed secondary (somatic) DMRs in paternally imprinted loci which are known to depend on the presence of a germline DMR and established after fertilization (Barlow and Bartolomei, 2014; Ferguson-Smith, 2011) and found that two tested regions sustained hypermethylation in TSa2i/L-treated cells (Fig. S3C).

**Figure 3:**
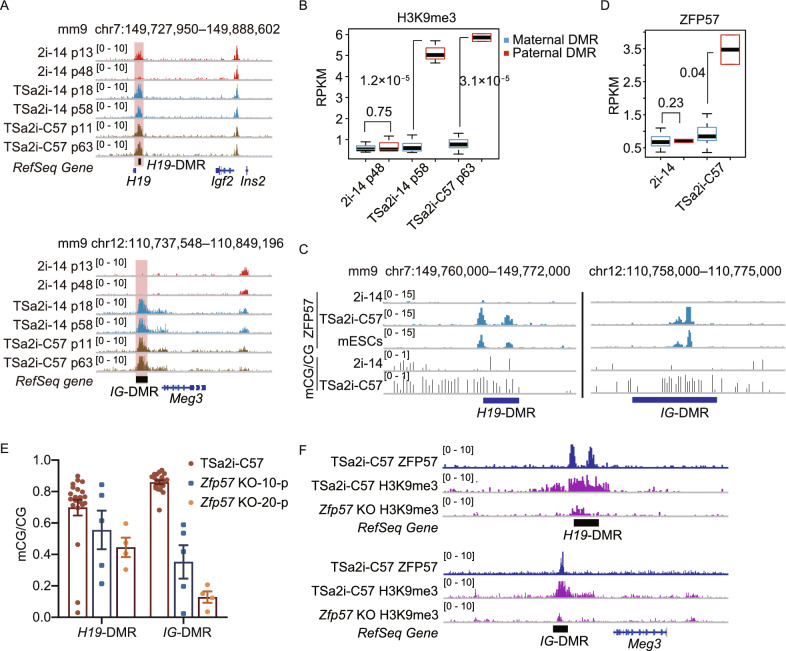
**H3K9me3 deposition and ZFP57 binding correlate with maintenance of paternal DMR methylation in TSa2i/L-derived AG-haESCs**. (A) Representative snapshots of H3K9me3 signals at *H19*-DMR and *IG*-DMR for the AG-haESCs derived in 2i/L (2i-14) and TSa2i/L (TSa2i-14 and TSa2i-C57) with different generations. (B) Boxplot showing H3K9me3 levels at maternal and paternal DMRs for 2i/L (2i-14) and TSa2i/L (TSa2i-14 and TSa2i-C57)-derived AG-haESCs. (C) Snapshots of ZFP57 binding signals and DNA methylation in *H19*-DMR and *IG*-DMR for the AG-haESCs derived in 2i/L (2i-14) and TSa2i/L (TSa2i-C57). ZFP57 ChIP-seq of mouse diploid ESCs (mESCs) is from the published data (Shi et al., 2019). (D) Boxplot showing ZFP57 levels at maternal and paternal DMRs for 2i/L (2i-14, p61) and TSa2i/L (TSa2i-C57, p63)-derived AG-haESCs. (E) The DNA methylation levels of *H19*-DMR and *IG*-DMR in wide-type AG-haESCs (TSa2i-C57) and the same AG-haESCs with *Zfp57* knockout culturing for 10 passages (*Zfp57* KO-10-p) and 20 passages (*Zfp57* KO-20-p). (F) Snapshot showing ZFP57 and H3K9me3 signals at *H19*-DMR and *IG*-DMR in wide-type and *Zfp57* KO TSa2i/L AG-haESCs

DNA methylation and H3K9 methylation are strongly associated (Du et al., 2015). In mouse ESCs cultured in 2i, while exhibiting globally hypomethylated DNA compared to ESCs maintained in conventional conditions involving serum, elevated DNA methylation correlates with the presence of H3K9me3 on imprinted loci (Habibi et al., 2013). Moreover, both parental genomes undergo large-scale H3K9me3 reestablishment after fertilization (Wang et al., 2018). We thus hypothesized that maintenance of hypermethylated germline DMRs in our sperm genome-bearing haploid cells may also correlate with the presence of H3K9me3, which is probably established during pre-implantation development of sperm-cloned embryos. To this end, we first analyzed the allelic H3K9me3 pattern on DMRs during mouse early embryo development based on single-nucleotide polymorphism (SNP) (Wang et al., 2018). As expected, both parental DMRs showed allelic-specific H3K9me3 patterns, which were established after fertilization and stably sustained from the zygote stage to the ICM stage (Fig. S4A and S4B). We then investigated the distributions of H3K9me3 in our TSa2i/L and 2i/L haploid cells that were derived from ICMs by performing ChIP-seq analysis. Consistent with a published report in mouse diploid ESCs (Walter et al., 2016), H3K9me3 was also mainly enriched at repetitive elements, especially LTRs and LINEs in haploid ESCs (Fig. S4C and S4D). Intriguingly, both *H19* and *IG* DMRs were stably enriched with strong H3K9me3 signals in TSa2i/L-derived AG-haESCs during long-term culturing (Fig. 3A). In contrast, H3K9me3 of paternal DMRs was gradually lost in 2i/L-derived AG-haESCs upon prolonged culture. Furthermore, H3K9me3 specifically marked paternal DMRs rather than maternal DMRs in TSa2i/L AG-haESCs, while all DMRs were free of H3K9me3 in 2i/L AG-haESCs (Figs. 3B and S4E). We also noted the loss of H3K9me3 at *Rasgrf1*-DMR in TSa2i-C57 cells with late passages (Fig. S4F), in which, *Rasgrf1*-DMR methylation was absent (Fig. S2C). Our results thus demonstrate that H3K9me3 of paternal DMRs is maintained in TSa2i/L AG-haESCs, which positively correlates with the DNA methylation state of DMRs.

ZFP57 is one of the Kruppel-associated box zinc finger proteins (KRAB-ZFPs) that can target and maintain genomic imprints after fertilization (Takahashi et al., 2015). Our sperm genome-carrying haploid cells were derived from androgenetic blastocysts, in which, paternal methylation imprints should be protected as those in normal diploid blastocysts (Li et al., 2008). Meanwhile, ZFP57 protein is highly expressed in ESCs, binds at all known imprinted DMRs in a parental origin-specific and methylation-sensitive manner and *Zfp57*-null ESCs lost imprints at all tested imprinted DMRs (Quenneville et al., 2011; Strogantsev et al., 2015). Together, these observations suggest that ZFP57 may be critical for maintenance of the paternal DMR methylation in TSa2i/L cells. As expected, ChIP-seq analysis indicated that methylated DMRs (in TSa2i/L cells) rather than unmethylated DMRs (in 2i/L cells) were bound by ZFP57 (Figs. 3C and S4G). Similarly, maternal DMRs were free of ZFP57 bindings in both 2i/L and TSa2i/L AG-mESCs (Fig. 3D). We then deleted *Zfp57* in TSa2i/L AG-haESCs and cultured them in SF/a2i/L for up to 20 passages (Fig. S4H). Expectedly, both *H19* and *IG* DMRs gradually lost methylation (Fig. 3E). Furthermore, WGBS analysis indicated that *Zfp57*-null cells were globally hypermethylated and sustained a reduced level of DNA methylations at paternal DMRs specifically (Fig. S4I). Consistently, H3K9me3 ChIP-seq analysis of *Zfp57*-null cells showed overt reduced signals on paternal DMRs (Fig. 3F). Taken together, TSa2i/L AG-haESCs preserve the epigenetic integrity of paternal imprints, including hypermethylated DNA methylation, deposition of H3K9me3 marks, and ZFP57 bindings.

### Deposition of H3K4me3 prevents *de novo* methylation of DMRs in 2i/L-SF/a2i/L-switched AG-haESCs

**Figure 4:**
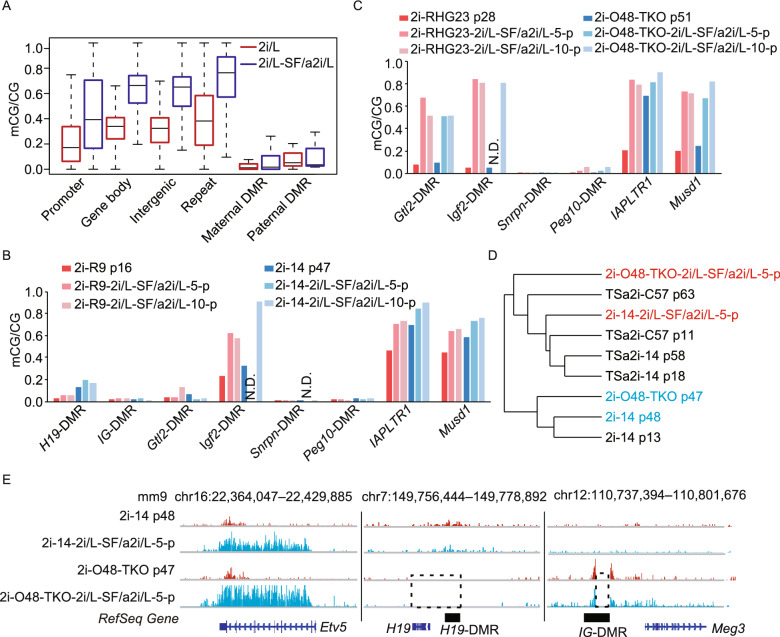
**Imprints cannot be restored in 2i/L-SF/a2i/L-switched AG-haESCs**. (A) Boxplot showing the DNA methylation levels of different gene elements in AG-haESCs (2i-14) cultured in 2i/L or switched to SF/a2i/L. (B) Bar plot showing the DNA methylation levels at germline and somatic DMRs for the 2i/L-cultured AG-haESCs (2i-R9 and 2i-14) and the same cells switched to SF/a2i/L for 5–10 passages. IAPLTR1 and Musd1 are two repeat elements. N.D., not detected. (C) Bar plot showing the DNA methylation levels at germline and somatic DMRs for the 2i/L-cultured AG-haESCs with both *H19* and *IG* DMR deletions (2i-RHG23 and 2i-O48-TKO) and the same cells switched to SF/a2i/L for 5–10 passages. IAPLTR1 and Musd1 are two repeat elements. N.D., not detected. (D) Hierarchical clustering analysis of H3K9me3 for the 2i/L, TSa2i/L, and 2i/L-SF/a2i/L switched AG-haESCs. (E) Snapshot of H3K9me3 levels at *Etv5*, *H19*-DMR, and *IG*-DMR for the AG- haESCs cultured in 2i/L or the same cells switched to SF/a2i/L. Deleted regions were labeled by dashed rectangles. 2i-O48-TKO cells were generated previously (Li et al., 2020)

Next, we sought to investigate whether imprints can be restored in 2i/L-cultured cells after being switched to SF/a2i/L conditions. Interestingly, switching 2i/L-treated haploid cells to SF/a2i/L for 5–10 passages induced global hypermethylation shown by 5mC immunoblotting (Fig. S5A), which was probably caused by the increased protein levels of DNA methyltransferases including DNMT1 and DNMT3A/3B (Fig. S5B). WGBS analysis further confirmed that while the whole genome underwent extensive *de novo* methylation in 2i/L-SF/a2i/L-switched AG-haESCs, both maternal and paternal DMRs maintained hypomethylated state (Fig. 4A). Bisulfite-PCR analysis further confirmed that paternal DMRs (*H19* and *IG* DMRs) and maternal DMRs (*Snrpn* and *Peg10* DMRs) did not undergo significant *de novo* methylation (Fig. 4B). Interestingly, when *H19* and *IG* DMR deletions existed in 2i/L-cultured haploid cells, the somatic DMRs (*Gtl2*-DMR and *Igf2*-DMR) were significantly methylated after being switched to SF/a2i/L culture conditions (Fig. 4C). Given that *Gtl2*-DMR and *Igf2*-DMR showed higher DNA methylation levels in TSa2i/L AG-haESCs when compared to 2i/L AG-haESCs (Fig. S3C), our results together further confirmed that establishment of somatic DMRs relies on germline-derived DMRs and deletion of germline-derived DMRs can partially mimic DMR methylation state (Edwards and Ferguson-Smith, 2007; Tucker et al., 1996). Although the global H3K9me3 signals were rescued with the global increase of DNA methylation (Figs. 4D and S5C), the absence of H3K9me3 signals on paternal DMR loci of 2i/L-cultured cells could not be restored after being switched to SF/a2i/L conditions, which was consistent with DNA methylation changes (Fig. 4E). Of note, ICAHCI analysis showed that switched cells even with global hypermethylation didn’t restore the developmental potential when germline DMRs were not established (Table S2). Meanwhile, when *H19* and *IG* DMR deletions existed in haploid cells, the switched cells displayed relatively high developmental potential similar to original 2i/L-treated DKO cells (Table S2). Collectively, these results indicate that imprints once lost can’t be restored in cultured ESCs under SF/a2i/L conditions, consistent with previous observations in other culture conditions (Tucker et al., 1996; Yagi et al., 2017).

**Figure 5:**
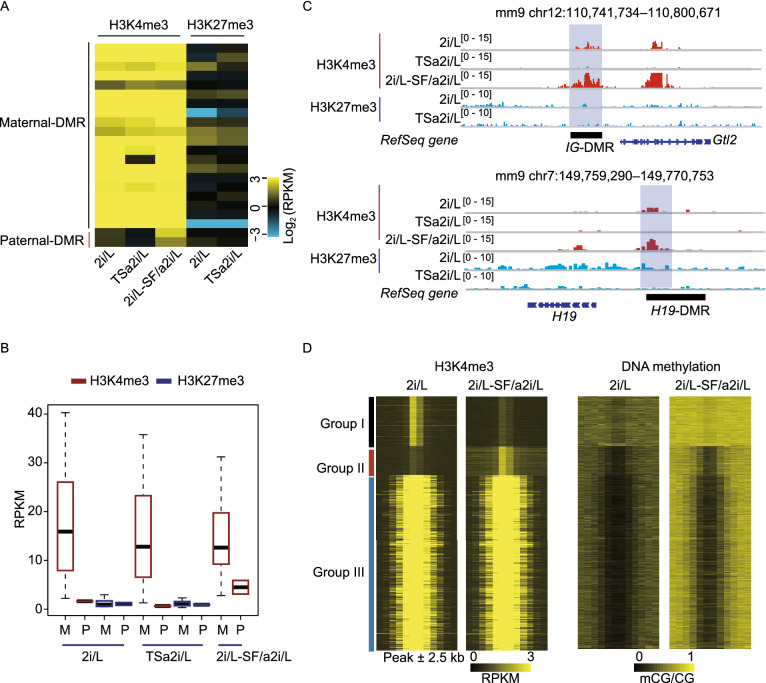
**Deposition of H3K4me3 prevents *de novo* methylation of hypomethylated germline DMRs**. (A) Heatmap representing the H3K4me3 and H3K27me3 levels of maternal and paternal DMRs in 2i/L (2i-14), TSa2i/L (TSa2i-14), and 2i/L-SF/a2i/L-switched (2i-14-2i/L-SF/a2i-10-p) AG-haESCs. (B) Bar plot showing H3K4me3 and H3K27me3 enrichment at maternal and paternal DMRs for 2i/L-cultured (2i-14), SF/a2i/L-cultured (TSa2i-14), and 2i/L-SF/a2i/L-switched (2i-14, switched from 2i/L to SF/a2i/L for 10 passages) AG-haESCs. (C) Snapshot of H3K4me3 and H3K27me3 at *IG*-DMR and *H19*-DMR for AG- haESCs cultured in 2i/L (2i-14), SF/a2i/L (TSa2i-14), or 2i/L-SF/a2i/L-switched (2i-14, 10 passages after swtiching) conditions. (D) Heatmap showing the H3K4me3 and DNA methylation levels within three groups of H3K4me3 peaks in 2i/L-cultured and 2i/L-SF/a2i/L-switched AG-haESCs. Each region was defined by extending H3K4me3 peaks for 2.5 kb upstream and downstream respectively

We next sought to reveal factors that are involved in preventing the *de novo* methylation at DMRs. Previous studies have shown that H3K4me3 is negatively correlated with DNA methylation that is associated with transcriptional activation and removing DNA methylation may lead to the spread of H3K27me3 to DNA that is otherwise protected from methylation (Atlasi and Stunnenberg, 2017), we thus analyzed the distributions of H3K4me3 and H3K27me3 in our cells. The results showed that nearly all unmethylated maternal DMRs were marked by H3K4me3 but not by H3K27me3 in 2i/L and TSa2i/L AG-haESCs (Fig. 5A and 5B). Meanwhile, paternal DMRs with hypomethylation also showed higher H3K4me3 enrichment in 2i/L-cultured AG-haESCs compared to TSa2i/L-cultured AG-haESCs with hypermethylated DMRs (Fig. 5C). Moreover, paternal DMRs showed enhanced H3K4me3 signals when AG-haESCs were switched from 2i/L to SF/a2i/L conditions (Fig. 5B and 5C). Consistently, high-throughput analyses showed that the loci with H3K4me3 peak lost during switching (Group I) were highly methylated in 2i/L-SF/a2i/L-switched cells, while the loci with maintained (Group III) or newly generated (Group II) H3K4me3 peaks were free of DNA methylation (Fig. 5D). Taken together, these results indicate that *de novo* methylation rigidly excludes the regions marked with H3K4me3. Due to the direct antagonism of H3K4me3 to DNMTs (Guo et al., 2015; Ooi et al., 2007), we proposed that H3K4me3 deposition at DMRs upon loss of both DNA methylation and H3K9me3 may prevent DMRs from *de novo* DNA methylation.

### Enhanced proliferation potential of AG-haESCs with hypermethylated *H19*-DMR accounts for increased DMR methylation in cells of late passages

**Figure 6:**
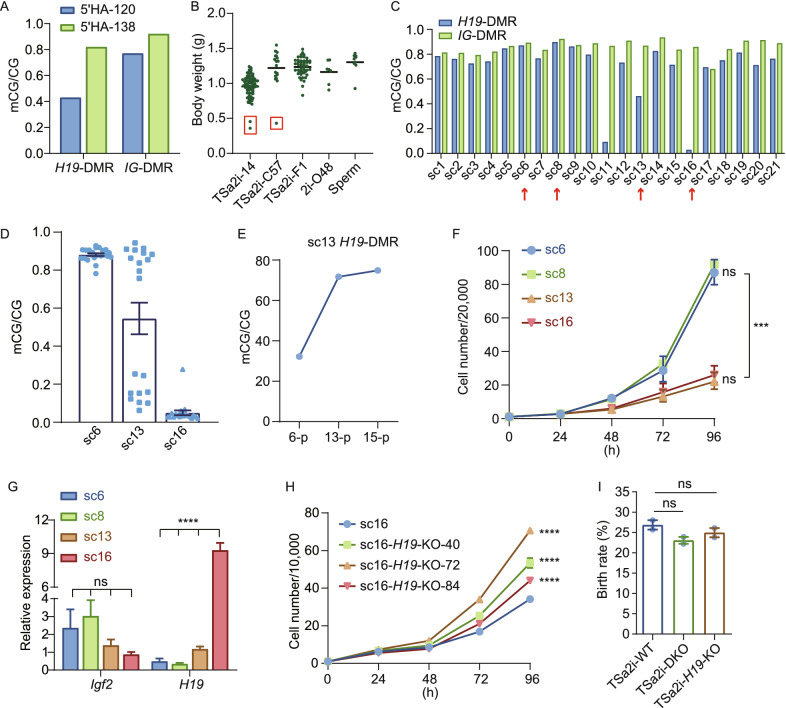
**Enhanced proliferation of cells with hypermethylated *H19*-DMR accounts for increased developmental potential of late-passage haploid cells**. (A) DNA methylation levels of *H19*-DMR and *IG*-DMR in the two *Dusp9*-HA-tagged AG-haESC lines (5’HA-120 and 5’HA-138) with distinct developmental potential. (B) The body weights of E18.5 SC pups produced by ICAHCI (from TSa2i-14, TSa2i-C57, and TSa2i-F1 AG-haESCs). Pups derived from DKO-AG-haESCs (2i-O48) and sperm are as controls. (C) DNA methylation levels of *H19* and *IG* DMRs in subclones from TSa2i-C57 (sc1 to sc21) were obtained through single-cell expansion. (D) DNA methylation levels of *H19*-DMR in the 2nd subclones expanded from single cells of the 1st subclones with high (sc6), middle (sc13), and low (sc16) levels of *H19*-DMR methylation. (E) Increased DNA methylation levels of *H19*-DMR in sc13 during culture. (F) Growth curve of the cells from subclones with high (sc6 and sc8), middle (sc13), and low (sc16) levels of *H19*-DMR methylation. ***, *P* < 0.0001. (G) Expression of *Igf2* and *H19* in sc6, sc8, sc13, and sc16 were analyzed by qPCR. ****, *P* < 0.00001; ns, not siginificant. (H) Growth curve of sc16-originated cells with hypomethylated *H19*-DMR and the same cells with *H19* KO (sc16-*H19*-KO-40, 72, or 84 representing three different KO lines). ****, *P* < 0.00001. I Birth rate of SC mice generated from TSa2i AG-haESCs (TSa2i-14, p60 and TSa2i-C57, p58), and TSa2i AG-haESCs with *H19* and *IG* DMR deletions (DKO-20, p68 and DKO-187, p68) or *H19*-DMR deletion (H19KO-40, p68-69). ns, not significant

Previous studies have shown that 2i/L-cultured AG-haESCs are a heterogeneous cell population regarding paternal DMR methylations, in which cells with different DNA methylation levels at *H19* and *IG* DMRs upon injection into oocytes result in different developmental potential of resulting SC embryos (Li et al., 2020; Yang et al., 2012; Zhong et al., 2015). Interestingly, two knockin haploid cell lines carrying *Dusp9*-HA established through single-cell expansion from the same experiment resulted in distinct differences in the birth rate of SC pups upon ICAHCI (32.5% vs. 8.3%, Table 1). As expected, higher levels of DNA methylation at *H19* and *IG* DMRs were indeed relative to a better birth rate (Fig. 6A). These observations implied that TSa2i/L-cultured haploid cells even with a high level of DMR methylations are still a heterogeneous population containing cells with different DNA methylation levels at *H19* and *IG* DMRs, leading to gene-modified cell lines with distinct DNA methylation and corresponding developmental potential. To confirm this, we first performed C-section at 18.5 days of gestation (E18.5) to recover a total of 111 SC pups originated from 111 single haploid cells of 3 cell lines, and found 3 growth-retarded pups (otherwise could not be obtained through natural delivery), which lost DNA methylation at *H19*-DMR as expected (Figs. 6B, S6A, and S6B). These results confirmed the heterogeneous methylome in TSa2i/L-cultured cells, excluding the roles of genome-editing manipulations in DMR methylation loss.

To further characterize the heterogeneity, we performed clonal expansion of single cells and bisulfite sequence analysis of *H19* and *IG* DMRs. From a total of 21 single-cell clones (1st clones), while three (sc11, sc13, and sc16) retained *H19*-DMR methylation at a reduced level, the rest clones sustained relatively higher methylation levels of *H19* and *IG* DMRs (Figs. 6C and S6C). These results suggested that most single cells carried hypermethylated *H19*-DMR and a small proportion of cells lost imprints during cell proliferation, consistent with a recent report that single-cell clonal expansion of diploid ESCs in S/L produces heterogeneous methylomes (Wang et al., 2020). In order to further determine the DNA methylation state of *H19*-DMR at single cell level of 1st clones, we performed secondary single-cell expansion using cells from 1st clones with hypermethylated (sc6), moderately-methylated (sc13), and hypomethylated (sc16) *H19*-DMR respectively. The results showed that the 2nd single-cell clones (2nd clones) from cells of 1st clones with hypermethylation or hypomethylation exhibited similar DNA methylation patterns at both DMRs (Fig. 6D), excluding the possibility of rigid DMR methylation changes in TSa2i/L-treated cells.

Interestingly, 2nd clones from cells of clone 13 were separated into two groups: one with hypermethylated *H19*-DMR and the other with hypomethylated *H19*-DMR (Fig. 6D). These results imply that a small proportion of cells displays more dynamically and less reliably at paternal DMRs, probably due to that DNMT1 has imprecise activity and H3K9me3-marked-region-related *de novo* activity during ESC division (Wang et al., 2020). Consistent with this postulation, *Dnmt1* mutation but not *Dnmt3a*/*3b* double knockout induced methylation loss at paternal germline DMRs in TSa2i/L AG-haESCs (Fig. S6D). We next investigated the changes of DNA methylation at paternal DMRs during prolonged culturing. To this, we expanded 1st clones with different methylation levels for 10 passages or more and analyzed DMR methylations. The results showed that clones with hypermethylation or hypomethylation overall maintained similar patterns (Fig. S6E). Consistently, in TSa2i/L-cultured cells that lost *H19*-DMR methylation, H3K9me3 signals were depleted, and as expected H3K4me3 marks were established (Fig. S6F). Interestingly, the sc13 with medium methylation of *H19*-DMR displayed increased methylation upon prolonged culture (Figs. 6E and S6E), consistent with our previous observations that late-passage cells sustained higher *H19*-DMR methylation level compared to early passages (Fig. 2B).

Finally, we attempted to determine what factors are involved in higher DMR methylation in TSa2i/L-treated haploid cells of late passages. Previous studies showed that androgenetic mouse embryonic fibroblasts (MEFs) display a shorter cell cycle compared with biparental MEFs, due to increased *Igf2* expression (Hernandez et al., 2003) and *H19* plays a physiological role in limiting the growth of the placenta before birth through suppresses cell proliferation (Keniry et al., 2012). As expected, cell proliferation analysis indicated that cell clones with *H19*-DMR hypermethylation displayed a better growth rate compared to cells with hypomethylation at *H19*-DMR (Fig. 6F). Of note, the sc13 with increased *H19*-DMR methylation after 10-passage expansion exhibited better proliferation (Fig. S6G). Consistently, cells with intact *H19*-DMR methylation had higher *Igf2* mRNA levels while cells with reduced *H19*-DMR methylation displayed increased *H19* expression (Fig. 6G). However, the perturbation of *Igf2* didn’t affect cell proliferation in clones with intact *H19*-DMR methylation (Fig. S6H and S6I). In contrast, deletion of *H19* induced increased cell proliferation in the clones with reduced *H19*-DMR methylation (Figs. 6H and S6J). We also characterized the self-diploidization of *H19* and *IG* DMR hyper- and hypo-methylated haploid cells and found that they displayed a similar diploidization rate (Fig. S6K). These results indicate that *H19* overexpression impairs the cell proliferation, implying that a better growth rate of haploid cells with *H19*-DMR hypermethylation may account for increased methylation levels in haploid cells of late passages under SF/a2i/L conditions.

Considering that a small fraction of TSa2i/L-treated cells carried hypomethylated *H19*-DMR that are related to the growth-retarded SC pups, we thus investigated whether removal of *H19*-DMR or both *H19* and *IG* DMRs in TSa2i/L-treated haploid cells may further improve their birth rate of SC pups through ICAHCI. However, DMR deletions did not improve the developmental potential of SC embryos (Table 1). In contrast, these deletions led to a slight reduction in the birth rate (Figs. 6I, S6L, and S6M), implying that original germline DMRs may sustain better developmental potential than DMR deletions. Moreover, these results further suggest that epigenetic integrity is critical for *in vivo* development. Taken together, our study suggest that *H19*- and *IG*-DMR methylation and H3K9me3 marks sustained in ICM cells are generally maintained during haploid ESC derivation and long-term expansion under TSa2i/L conditions; and epigenetic balance may be achieved during cell proliferation through joint roles of enhanced division rate by cells with intact *H19*-DMR methylation and a small proportion of cell gradually losing paternal DMR methylation due to imprecise DNMT1 activity-induced epimutation (Wang et al., 2020).

## DISCUSSION

The breakthrough of the derivation of mouse ESCs from embryos was made in 1981 under the conditions containing feeder layers and serum (Evans and Kaufman, 1981). Following studies identified that the cytokine LIF produced by feeder cells is the principal factor to support ESC self-renewal, which can replace feeders in both derivation and long-term culture of germline-competent ESCs (Martello and Smith, 2014). Nonetheless, S/L conditions yield overt cellular heterogeneity, consisting of a substantial number of differentiating cells. Meanwhile, there are nonpermissive strains of mice for ESC derivation using S/L. Moreover, while early-passage ESCs under S/L can develop to all ESC-derived fetuses upon injection into tetraploid blastocysts, upon prolonged culturing the developmental potential of many ESC lines becomes impaired due to epigenetic alterations in imprinted genes (Dean et al., 1998; Nagy et al., 1990, 1993; Wang et al., 1997). The advent of 2i, however, not only enables efficient and reliable derivation of ESC lines from all mouse strains (Kiyonari et al., 2010; Nichols et al., 2009) but also establishes ground state pluripotency with robust self-renewal of a biologically homogeneous population of cells (Ying et al., 2008). Nevertheless, two recent studies have shown that prolonged culture of ESCs in 2i conditions induces a widespread loss of DNA methylation, leading to the impaired developmental potential of ESCs (Choi et al., 2017b; Yagi et al., 2017). In both reports, a2i/L (Shimizu et al., 2012) was employed to preserve the epigenetic integrity as well as the developmental potential of ESCs. However, one study showed that prolonged culture of female ESCs in a2i/L resulted in a reduction of germline DMR methylation although with increased global methylation (Yagi et al., 2017). Meanwhile, stable maintenance of DMR methylation in XY ESCs has been reported only to passage 15, leaving an intriguing question whether germline DMR methylation can be stably maintained in mouse ESCs during long-term culturing. Although recent studies have been examining the maintenance of genomic imprints in diploid ESC under different media (Lee et al., 2018; Wu et al., 2020, 2021), an ESC line with stable imprints during long-term culture is still not established. In our study, we generated multiple haESC lines from androgenetic blastocysts of different strains with stably hypermethylated *H19* and *IG* DMRs up to passage 60 using a reported two-step derivation/culture protocol with a2i/L (Yagi et al., 2017). These cells result in efficient generation of SC mice through ICAHCI. Strikingly, in contrast to previous observations in mouse ESCs, our TSa2i/L-treated AG-haESCs have increased paternal DMR methylation and improved developmental potential upon prolonged culture.

**Figure 7:**
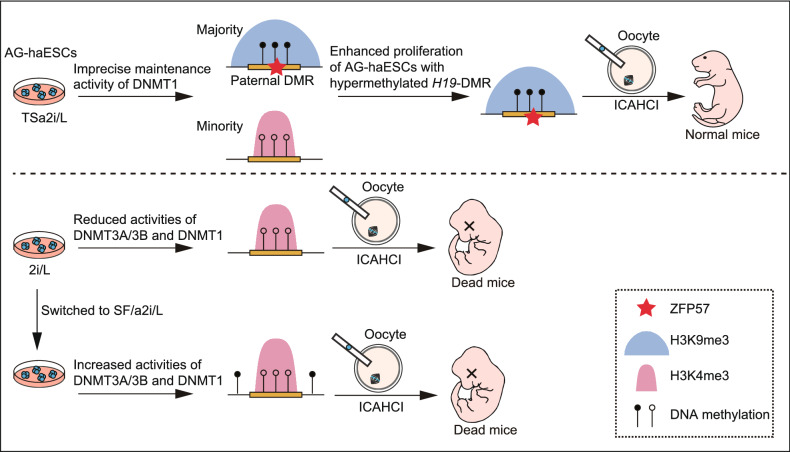
**A model showing that epigenetic integrity of paternal DMRs enhances the developmental potential of AG-haESCs**. Schematic of epigenetic features of paternal DMRs in TSa2i/L-treated AG-haESCs (upper) and 2i-SF/a2i-switched AG-haESCs (bottom). Open and filled circles represent unmethylated and methylated CpG sites, respectively

TSa2i/L conditions ensure the dynamic equilibrium of typical paternal epigenetic makeups and improved sperm-like features without compromising genetic integrity in AG-haESCs. Multiple factors may be involved in this epigenetic balance, including: (1) the coexistence of DNA methylation and H3K9me3 marks and ZFP57 bindings ensures stable maintenance of paternal imprints; (2) the imprecise DNA methylation of DNMT1 may cause methylation loss at paternal DMRs in a small number of cells; (3) H3K4me3 deposition after loss of DNA methylation and H3K9me3 marks precludes *de novo* methylation at germline DMRs, and (4) cells with hypermethylated *H19*-DMR have enhanced proliferation potential compared to cells with hypomethylated *H19*-DMR (Fig. 7). In addition, haploid cells cultured in a2i/L with serum lost paternal imprints in late passages, which indicates that serum may result in loss of DMR methylation during prolonged culturing. Considering that our haploid cells were always maintained on feeder cells, we also wanted to know if feeder cells are an important factor for maintenance of imprints in our SF/a2i/L conditions. Although short-term culture without feeders didn’t change the DMR methylations (Fig. S7), feeder cells may probably release important but unknown factors that contribute to paternal imprint maintenance during long-term culturing. Moreover, DNMT1 has H3K9me3-marked-region-related *de novo* activity during ESC division (Wang et al., 2020), which may also play a role for the increased *H19*-DMR methylation in late-passage haploid cells. Future studies will be needed to understand the detailed mechanism underlying the long-term stable maintenance of paternal imprints through simplifying the culture conditions, such as using a feeder-free system.

Thus, TSa2i/L-treated AG-haESCs serve as a unique ESC system for imprinting control analysis *in vitro*. Importantly, in combination with ICAHCI technology, these analyses can be promoted to the organismal level in one step (Li and Li, 2019). Unlike mature sperm or round spermatids, a small proportion of TSa2i/L-treated AG-haESCs lost *H19*-DMR methylation during cell division. While DNMT1-mediated imprecise DNA methylation may partially account for this loss (Wang et al., 2020), the detailed mechanisms require further elucidation. Moreover, *Rasgrf1*-DMR methylation is stably maintained in two cell lines but dramatically lost in the other one at late passages. Further investigation into the underlying difference may help us to understand how *Rasgrf1*-DMR methylation is controlled. These comparative studies may also yield clues to further define conditions for stable maintenance of *Rasgrf1* imprinting *in vitro*, which may facilitate the potential applications of these cells. One intriguing application is the derivation of PG-haESCs carrying oocyte genome that can long-term maintain methylation of the majority of maternal DMRs.

## METHODS

### Animals

All mice were housed in individually ventilated cages (IVC) under specific pathogen-free conditions and a 12:12 h light/dark cycle. MII oocytes were all from B6D2F1 (C57BL/6♀ × DBA/2♂) female mice. All the pseudopregnant foster mothers were ICR females. All animal procedures were performed under the ethical guidelines of the Shanghai Institute of Biochemistry and Cell Biology, Chinese Academy of Sciences, Shanghai, China.

### Derivation of AG-haESCs

Mature oocytes were obtained from superovulated B6D2F1 (C57BL/6 × DBA2) female mice and enucleated in a droplet of HEPES-CZB medium containing 5 μg/mL cytochalasin B (CB) using a blunt Piezo-driven pipette. After enucleation, a single sperm head was injected into oocyte cytoplasts. The reconstructed oocytes were cultured in the CZB medium for 1 h and then activated for 5–6 h in the activation medium containing 10 mmol/L Sr^2+^. Following activation, all the reconstructed embryos were cultured in the KSOM medium with amino acids at 37 °C under 5% CO_2_ in the air. The reconstructed embryos that reached the blastocyst stage by 4.5 days were used for derivation of ESC lines. The zona pellucida was removed using acid Tyrode solution. Each blastocyst was transferred into one well of a 96-well plate seeded with ICR embryonic fibroblast feeders in different ESC media. For the establishment of S/L haESCs, the culture media was DMEM (MILLIPORE), 100× NEAA (MILLIPORE), 100× Nucleosides (MILLIPORE), 100× L-Glutamine (MILLIPORE), 100× P/S (GIBCO), 100× β-mercaptoethanol (MILLIPORE), 15% FBS and 1,000 U/mL LIF. For the establishment of 2i/L and a2i/L haESCs, the culture medium was DMEM, 100× NEAA, 100× Nucleosides, 100× L-Glutamine, 100× P/S, 100× β-mercaptoethanol, 15% FBS and 1,000 U/mL LIF which was supplemented with 1 μmol/L PD0325901 (SELLECK) and 3 μmol/L CHIR99021 (SELLECK) for 2i/L and 1.5 μmol/L CGP77675 (SIGMA) and 3 μmol/L CHIR99021 for a2i/L. For the derivation of TSa2i/L haESCs, a blastocyst was cultured in a2i/L medium firstly. After two passages, the cells were maintained in SF/a2i/L medium containing DMEM/F12 (GIBCO) and Neurobasal (GIBCO) supplemented with B27 (GIBCO) and N2 cell-supplements (GIBCO), 100×P/S, 2 mmol/L Glutamax (GIBCO), and 1,000 U/mL LIF, 1.5 μmol/CGP77675 and 3 μmol/L CHIR99021. To enrich haploid cells, ESCs were trypsinized, washed by DPBS (GIBCO), and then incubated with 15 mg/mL Hoechst 33342 in a 37 °C water bath, followed by fluorescence-activated cell sorting (FACS, BD FACS AriaII) for 1C peak.

### CRISPR-Cas9-mediated gene manipulation in haploid cells

To generate CRISPR-Cas9 plasmids for gene mutation, sgRNAs (Table S3) of target genes were synthesized, annealed, and ligated to the pX330-*mCherry* plasmids (Wu et al., 2013) that were digested with BpiI (Thermo Scientific). AG-haESCs were transfected with the corresponding pX330-*mCherry* plasmids using Lipofectamine 3000 (Life Technologies) following the manufacturer’s instruction manual. 24 h after transfection, the haploid cells expressing red fluorescence protein were enriched by flow cytometry (FACS AriaII, BD Biosciences) and plated at low density. Six days after plating, single colonies were picked for the derivation of AG-haESCs.

### Intracytoplasmic AG-haESC or sperm injection and embryo transfer

To generate SC embryos, AG-haESCs were treated with 0.05 μg/mL Demecolcine solution (Sigma, USA) for 10–12 h and synchronized to the M phase. Next, the M-phase clones were treated with trypsin and suspended in the HCZB medium. MII oocytes were collected from oviducts of superovulated B6D2F1 females (8 weeks old). AG-haESCs were injected into the cytoplasm of MII oocytes in a droplet of HCZB medium containing 5 μg/mL cytochalasin B (Sigma, USA) using a Piezo-drill micromanipulator. The injected oocytes were cultured in the CZB medium for 30 min and then activated for 5–6 h in Ca^2+^ free CZB with SrCl_2_. For sperm injection, the procedures were the same as described above. Following activation, the reconstructed embryos were cultured in AA-KSOM (Merk, Germany) medium at 37 °C under 5% CO_2_ in the air. The embryos obtained through Intracytoplasmic AG-haESC Injection (ICAHCI) were cultured in KSOM medium for 24 h to reach the two-cell stage. Thereafter, 18–20 two-cell embryos were transferred into each oviduct of pseudopregnant ICR female mice at 0.5 dpc. Recipient mothers carrying SC embryos derived from 2i/L or a2i/L medium were euthanized at 18.5 or 19.5 days of gestation, and the pups were quickly removed from the uteri.

### RNA extraction and Real-time quantitative PCR

Total RNA was isolated from the cells using TRIzol reagent (Invitrogen). The cDNA was obtained from about 1 μg RNA with a reverse transcription reaction by the ReverTra Ace qPCR RT Master Mix (TOYOBO). Real-time quantitative PCR reactions (RT-qPCR) were performed on a Bio-Rad CFX96 using the SYBR Green Mix (TOYOBO) in triplicate. All the gene expression levels were normalized to the internal standard gene *Gapdh* or *β*-*actin*. The primer sequences are listed in Table S3.

### Bisulfite PCR

The genome extraction from ESCs used the TIANamp Genomic DNA Kit (TIANGEN, China). RNA was removed using RNaseA (Thermo Scientific) in a 37 °C water bath for 1 h. DNA was recovered using a Universal DNA Purification Kit (TIANGEN, China). The bisulfite conversion was performed using the EZ DNA Methylation-Gold TM Kit (ZYMO research, USA) for 200–500 ng genomic DNA, following the manufacturer’s instructions. The bisulfite DNA products were amplified by nested PCR. PCR primers are listed in Table S3. The amplified products were purified by gel electrophoresis using a Universal DNA Purification Kit and cloned into pMD™19-T Vector Cloning Kit (Takara, 6013). For each sample, more than 12 *E*. *coli* clones were picked for sequencing. The results were analyzed by the DNA methylation analysis platform. (http://services.ibc.unistuttgart.de/BDPC/BISMA/). To detect DNA methylation in multiple regions with high efficiency, we mixed all bisulfite PCR products from one sample and performed the high-throughput sequencing library preparation described later. The sequencing reads were mapped to mm9 using BSMAP with parameters –r 0 –w 100 –v 0.1 –A AGATCGGAAGAGC. Methylation level was calculated using methylated read number versus total read number for one CpG site. For each bisulfite PCR region, the average DNA methylation level of all CpG sites in it was calculated.

### Whole-genome bisulfite sequence (WGBS)

A total amount of 5–10 μg genomic DNA was mixed with 25 ng lambda DNA and sonicated to 200–500 bp, followed by end repair and dATP adding with the homemade Kit. Next, methylated adapters (synthesized by ThermoFisher Scientific) were ligated to the sonicated DNA. Ampure beads (Vazyme) were used to remove <200 bp fragments. Bisulfite treatment was performed using the EZ DNA Methylation-Gold TM Kit (ZYMO research, USA). After bisulfite conversion, the single-stranded, uracil-containing DNA was subjected to 10–12 cycles of PCR reaction with Illumina TruSeq PCR primers and 2.5 U of *Pfu Turbo* *C*_*x*_ Hotstart DNA polymerase (Agilent Technologies) to recover enough DNA for sequencing. The sequencing reads were aligned to mm9 using BSMAP with parameters –r 0 –w 100 –v 0.1 –A AGATCGGAAGAGC. Multiple mapped reads and PCR duplicates were removed. After mapping, those reads with total CG coverage less than 5 within 200 bp were removed. The methylation level was calculated using methylated CpG versus total CpG in each bin. The file with DNA methylation levels for each 200 bp bin was used for further analysis. For the boxplots about DNA methylation, we calculated the average DNA methylation levels of each region for each kinds of gene elements. The genomic information of maternal DMRs (*n* = 20) and paternal DMRs (*n* = 3) used in the WGBS analysis were shown in Table S4.

### ChIP-seq

Cells were collected and cross-linked with 1% formaldehyde. Cells were then suspended in cell lysis buffer (0.3% SDS, 50 mmol/L Tris-HCl pH 8.0, 20 mmol/L EDTA, and freshly added protease inhibitors) and incubated for 10 min on ice followed by sonication. About 30–60 μg of fragmented chromatin was diluted in dilution buffer (16.7 mmol/L of Tris-HCl, pH 8.0, 1.1% Triton X-100, 1.2 mmol/L of EDTA, 167 mmol/L of NaCl, freshly added protease inhibitor) and then incubated with 5 μg antibody at 4 °C for 6–8 h. Next, the pretreated Dynabeads (catalog no. 11201D; Invitrogen) were incubated with chromatin and antibody mixture overnight at 4 °C. Then, Dynabeads were washed with washing buffer (50 mmol/L of HEPES, pH 8.0, 1% NP-40, 0.7% deoxycholate, 0.5 mol/L of LiCl, freshly added protease inhibitor) 5 times followed by washing with Tris-EDTA buffer (10 mmol/L of Tris-HCl, pH 8.0, 0.1 mmol/L of EDTA) once. Then, 100 μL of elution buffer (50 mmol/L of Tris-HCl, pH 8.0, 1 mmol/L of EDTA, 1% SDS) was added and Dynabeads were incubated in ThermoMixer at 65 °C for 30 min at maximum speed. The supernatant was collected and treated with proteinase K at 55 °C for 2 h, then purified with the TIANquick Mini Purification Kit (catalog no. DP203-02; Tiangen). RNA was removed by treatment with RNase at 37 °C for 1 h. DNA was then purified by AMPure beads (Vazyme) and subjected to DNA library preparation as described later on. ChIP-seq reads were aligned to mm9 with Bowtie2 (version 2.2.2) with parameters -t -q -N 1 -L 25. All unmapped reads, multiply mapped reads and PCR duplicates were removed. To generate the ChIP-Seq signals for each histone modification shown in the UCSC genome browser, we normalized the read counts by computing the number of reads per kilobase of bin per million reads sequenced (RPKM). The RPKM file was used to generate genome browser snapshots and further analysis. The used antibodies included Histone H3K9me3 antibody (Active Motif, Cat. No. 39161, Lot. No. 09919003), Histone H3K4me3 antibody (MERCK, Cat. No. 04-745, Lot. No. 3068440), Histone H3K27me3 antibody (Diagenode, Cat. No. C15410069, Lot. No. A1818P) and ZFP57 antibody (Abcam, Cat. No. ab45341, Lot. No. GR3270465-1).

### High-throughput sequencing library preparation and sequencing

DNA was end-repaired, adenylated, and ligated to TruSeq sequencing adapters. After purification with VAHTS DNA Clean Beads (Vazyme), DNA was amplified by Phusion High-Fidelity DNA Polymerase (catalog no. M0530L; New England Biolabs) or *Pfu Turbo C*_*x*_ Hotstart DNA polymerase (for bisulfite-treated DNA libraries). The amplified DNA was size-selected using 2% agarose gel or VAHTS DNA Clean Beads for 200 to 500-bp DNA fragments. All libraries were sequenced using an Illumina HiSeq 2500 or X10 system according to the manufacturer’s instructions.

### RNA-seq

500–1000 haploid cells were calculated by trypan blue staining. Amplificated cDNAs were collected by Single-Cell Full Length mRNA-Amplification Kit (Vazyme) according to the manufacturer’s instructions. RNA-seq libraries were prepared by TruePrep DNA Library Prep Kit V2 for Illumina (Vazyme) according to the manufacturer’s instructions. All RNA-seq reads were mapped to mm9 with TopHat (version 2.2.1). The mapped reads were further analyzed by Cufflinks, and the expression levels for each transcript were quantified as Fragments Per Kilobase of transcript per Million mapped reads (FPKM).

### 5mC dot blot

DNA samples diluted to the defined concentration were treated with 0.4 mol/L of NaOH and 10 mmol/L of EDTA at 99 °C for 10 min before being cooled on ice immediately. The denatured DNA was loaded onto a nylon transfer membrane (catalog no. RNP303B; GE Healthcare) followed by ultraviolet cross-linking. The membrane was air-dried and blocked in a blocking buffer (10% milk, 1% BSA in PBS with Tween 20 (PBST)) at room temperature for 1 h. 5mC antibody (1:1000 dilution; Eurogentec, BI-MECY-1000) was diluted in a blocking buffer and incubated with the membrane at room temperature for 3 h. The membrane was washed with 1× PBST and incubated with a secondary antibody at room temperature for another 1 h. Then, the membrane was incubated with enhanced chemiluminescence substrate for exposure after washing with 1× PBST.

### Western blot

Proteins were separated by SDS–PAGE and then blotted on a PVDF (polyvinylidene difluoride) membrane, blocked with blocking solution (5% non-fat dry milk in TBST buffer) for 1 h and incubated with the appropriate primary antibody in blocking solution overnight at 4 °C. The membranes were washed three times for 10 min each with TBST and incubated with the appropriate secondary antibody in blocking solution for 1 h at room temperature. Chemiluminescence detection was performed using an ECL Western Blotting Detection kit from GE Healthcare. The used antibodies included anti-DNMT1 (1:2,000 dilution; Cell Signaling Technology, 5032S), anti-UHRF1 (1:2,000 dilution; Santa Cruz, sc-98817), anti-DNMT3A (1:2,000 dilution; Proteintech, 20954-1-AP), anti-DNMT3B (1:2,000 dilution; Abcam, ab2851), anti-DNMT3L (1:2,000 dilution; Santa Cruz, sc-393603), anti-ZFP57 (1:2,000 dilution; Abcam, ab45341), and anti-ACTB (1:10,000 dilution; Sigma, A5441).

## Supplementary Information

Below is the link to the electronic supplementary material.

Supplementary file1 (PDF 11708 kb)

## Declarations

This study was supported by Genome Tagging Project and grants from the Chinese Academy of Sciences, the National Key Research and Development Program of China, and the National Natural Science Foundation of China (2019YFA0109900, 2020YFA0509000, XDB19010204, QYZDJ-SSW-SMC023, Facility-based Open Research Program, 31821004, 32030029, and 31730062). The research is partly supported by the Fountain-Valley Life Sciences Fund of University of Chinese Academy of Sciences Education Foundation.

The raw data of RNA-seq, ChIP-seq, WGBS, and processed data in this study were deposited at Gene Expression Omnibus (GEO) with accession numbers GSE168447.

J.L and H.Z conceived of the project. H.Z and Y.M derived the haploid cells. Y.M and Q.Y performed the ICAHCI and ICSI experiments. H.Z, Y.M and C.L performed the molecular biological experiments. Y.L and H.Z performed RNA-seq, ChIP-seq and WGBS. Y.L analyzed the high-throughput data. J.L. supervised the project and wrote the manuscript.
