# Supplementary material for: Epigenetic integrity of paternal imprints enhances the developmental potential of androgenetic haploid embryonic stem cells
Source: Protein Cell. 2021 Dec 5;13(2):102–19. doi: 10.1007/s13238-021-00890-3 (PMC8783938; doi:10.1007/s13238-021-00890-3)
Supplement: Supplementary file 1 — Supplementary file1 (PDF 11708 kb) [file 13238_2021_890_MOESM1_ESM.pdf]

Supplementary Tables

Table S1. Summary of *in vivo* development of ICAHCI embryos derived from haploid cells in different media.

Medium	Cell line	Passage number	No. of transferred embryos	No. of growth-retarded pups (% of transferred embryos)	No. of normal pups (% of transferred embryos)
2i/L	2i-12	p9	216	2 (0.9)	2 (0.9)
	2i-14	p31	108	0	0
	2i-14	p54	80	0	0
	**Subtotal**	**p9-p54**	**404**	**2 (0.5)**	**2 (0.5)**
a2i/L	a2i-8	p9	36	1 (2.7)	4 (11.1)
	a2i-8	p40	90	0	5 (5.5)
	a2i-8	p54	120	0	6 (5)
	**Subtotal**	**p9-p54**	**246**	**1 (0.4)**	**15 (6.1)**

All pups were delivered by C-section at E19.5

Table S2. Summary of SC mice derived from 2i/L and 2i/L-SF/a2i/L-switched AG-haESCs.

Genotype	Cell line	Medium	Passage number	No. of transferred embryos	No. of growth-retarded pups (% of transferred embryos)[e]	No. of normal pups (% of transferred embryos)[e]
Wild-type	2i-7	2i/L	p37	117	1 (0.9)[e]	0[e]
		SF/a2i/L	14-p[d]	126	0[e]	0[e]
	2i-14	2i/L	p47	80	0[e]	0[e]
		SF/a2i/L	10-p[d]	200	1 (0.5)[e]	0[e]
	**Subtotal**	**2i/L**		**197**	**1 (0.5)[e]**	**0[e]**
		**SF/a2i/L**		**326**	**1 (0.3)[e]**	**0[e]**
H19-DMR & *IG*-DMR knockout	O48[a]	2i/L	p51	140		19 (13.6)
		SF/a2i/L	14-15-p[d]	525		89 (17)
	RHG23[c]	2i/L	p28	105		16 (15.2)
		SF/a2i/L	9-p[d]	140		18 (12.9)
	RHG24[c]	2i/L	p28	105		19 (18.1)
		SF/a2i/L	9-p[d]	105		15 (14.3)
$H19^{\Delta 13Kb}$ & *IG* DMR knockout	O48 TKO[b]	2i/L	p51-52	350		57 (16.3)
		S/Fa2i/L	9-10-p[d]	280		39 (13.9)
	**Subtotal**	**2i/L**		**700**		**111 (15.9)**
		**SF/a2i/L**		**1050**		**161 (15.3)**

a: Generated previously (Zhong et al., 2015).
b: Generated previously (Li et al., 2020).
c: Generated in this study.
d: 2i/L-cultured AG-haESCs switched to SF/a2i/L medium for different passages.
e: Delivered by C-section at E18.5.

Table S3. List of primers and sgRNA sequences in this study.

Primer Name	Sequence (5'-3')	Application
H19-BS-OF	GAGTATTTAGGAGGTATAAGAATT	Bisulfite sequencing
H19-BS-OR	ATCAAAAACTAACATAAACCCCT	
H19-BS-IF	GTAAGGAGATTATGTTTATTTTTGG	
H19-BS-IR	CCTCATTAATCCCATAACTAT	
IG-BS-OF	TTAAGGTATTTTTTATTGATAAAATAATGTAGTTT	
IG-BS-OR	CCTACTCTATAATACCCTATATAATTATACCATAA	
IG-BS-IF	GATCTCGAGCTCAAGCTTCGCTATAATTTATCATAAACAAATCCCATAACTTACT	
IG-BS-IR	CAGTTATCTAGATCCGGTGTTAGGAGTTAAGGAAAAGAAAGAAATAGTATAGT	
Gtl2-BS-OF	AAAGGTTAGTGTTGGGGATTT	
Gtl2-BS-OR	TCTAAATTCAAAATTACTAATCAACA	
Gtl2-BS-IF	TGTAAGGAAAAGAATTTTTAGGTA	
Gtl2-BS-IR	AAAAAACTTTCAACCACCAAAAC	
Igf2-BS-OF	GATTTTTGGAGGGTAGAAAGTAGAGAT	
Igf2-BS-OR	TATCCCATTCCAAAAACATCTAAC	
Igf2-BS-IF	TGAAATAGGTTATAGGTTGTGA	
Igf2-BS-IR	ATCCCTAACTTTTCTAACCTC	
Snrpn-BS-OF	TATGTAATATGATATAGTTTAGAAATTAG	
Snrpn-BS-OR	AATAAACCCAAATCTAAAATATTTTAATC	
Snrpn-BS-IF	AATTTGTGTGATGTTTGTAATTATTTGG	
Snrpn-BS-IR	ATAAAATACACTTTCACTACTAAAATCC	
Peg10-BS-OF	GTATTTAATTTGGAAAGTTGTAGGAGAG	
Peg10-BS-OR	TACAACAAAAATAAATCCCCCACCTC	
Peg10-BS-IF	GGAAAGTTGTAGGAGAGTAATTAAA	
Peg10-BS-IR	ACAAACTATTACTAAACACCCATTC	
Musd1-BS-OF	AAATTTGAGTTTTGATTAGTATGAAATTGT	
Musd1-BS-OR	AATCTAATATTTCTTCTTCCTTAAACCATA	
Musd1-BS-IF	AAATTTGAGTTTTGATTAGTATGAAATTGT	

Musd1-BS-IR	AACTTTAAACCCTTTCTTCTTCCACCTAAA	
IAP-BS-OF	TTGATAGTTGTGTTTTAAGTGGTAAATAAA	
IAP-BS-OR	CAAAAAAAACACCACAAACCAAAAT	
IAP-BS-IF	TTGTGTTTTAAGTGGTAAATAAATAATTTG	
IAP-BS-IR	AAAACACCACAAACCAAAATCTTCTAC	
H19-qPCR-F	TGTAAACCTCTTTGGCAATGCTGCC	Realtime PCR
H19-qPCR-R	TATTGATGGACCCAGGACCTCTGGT	
Igf2-qPCR-F	CTAAGACTTGGATCCCAGAACC	
Igf2-qPCR-R	GTTCTTCTCCTTGGGTTCTTTC	
Gtl2-qPCR-F	TTGCACATTTCCTGTGGGAC	
Gtl2-qPCR-R	AAGCACCATGAGCCACTAGG	
Dlk1-qPCR-F	ACTTGCGTGGACCTGGAGAA	
Dlk1-qPCR-R	CTGTTGGTTGCGGCTACGAT	
Gapdh-qPCR-F	CACTCTTCCACCTTCGATGC	
Gapdh-qPCR-R	CTCTTGCTCAGTGTCCTTGC	
H19-DMR-CHIP-F	GCACAGCGTGGAGAGTGAAC	
H19-DMR-CHIP-R	CATTTCTTGGGTAGCTCCTTCAG	
IG-DMR-CHIP-F	TGGGTTTACCGTAAAGGATGATTT	
IG-DMR-CHIP-R	CTCTGGCACAAAGCAATGATACA	
Rasgrf1-CHIP-F	GCTGCTGCCGCTAAAGATAG	
Rasgrf1-CHIP-R	CAGCGCTGGCTTTATAAACTCTC	
Actb-CHIP-F	CGTATTAGGTCCATCTTGAGAGTAC	
Actb-CHIP-R	GCCATTGAGGCGTGATCGTAGC	
Olfr9-CHIP-F	CAACATCCCAGGGTCGTCA	
Olfr9-CHIP-F	CTGGTCTGTGTTCCCAAATGAATC	
Tet1-F	AGGTGCATAGTGGGAGCCTA	Sequencing of *Tet1* mutation
Tet1-R	GAAGACTTGGCTGAACCGGA	
Tet2-F	AGATCTCCTCTCCAAGCCGT	Sequencing of *Tet2* mutation
Tet2-R	TGGATCCCAGACTCCAGCTT	
Tet3-F	CAGGTGGAACAGGAGCAGAG	Sequencing of *Tet3* mutation
Tet3-R	GGTCCCGTGATGGTGAATGT	
Dusp9-F	TCAGGACAGGGGTTGACTTC	Sequencing of *Dusp9* mutation
Dusp9-R	CTGGTCGTACAGGAGCACAG	
H19-KO-F	GTGGTTAGTTCTATATGGGG	Genotyping of *H19*-DMR knockout
H19-KO-R	TCTTACAGTCTGGTCTTGGT	
IG-KO-F	TGTGCAGCAGCAAAGCTAAG	Genotyping of

IG-KO-R	ATACGATACGGCAACCAACG	*IG*-DMR knockout
Dnmt1-KO-F	AAGCATCAGGTGTCAGAGCC	Genotyping of *Dnmt1* knockout
Dnmt1-KO-R	TGGTCCCAGCTACCCGATTA	
Dnmt1-WT-F	GCGATGTGGAGATGCTGTGT	
Dnmt1-WT-R	GAGGCCATTTCTGTCCCTCTG	
Dnmt3a-KO-F	TCCACTGGGCCTAATGCAAC	Genotyping of *Dnmt3a* knockout
Dnmt3a-KO-R	CTCCCGCTGAGAACTACAGG	
Dnmt3a-WT-F	ACACCCACTTTGGTAGGTCC	
Dnmt3a-WT-R	TGTTCCCACACATGAGCACT	
Dnmt3b-F	AAGTGCAAACAGGGCAGTT	Sequencing of *Dnmt3b* mutation
Dnmt3b-R	TGTTGTGTCTGGGAAGGACC	
H19-gene-KO-F	GCCTCGGGAGTTGGGATTAG	Genotyping of *H19* gene knockout
H19-gene-KO-R	GAGCAAAGGCATCGCAAAGG	
H19-gene-WT-F	GCTCACCAAGAAGGCTGGAT	
H19-gene-WT-R	ACACTGTATGCCCTAACCGC	
Igf2-KO-F	GGGGTGTCAATTGGGTTGTTT	Genotyping of *Igf2* knockout
Igf2-KO-R	GGGATTAGGGGTGTGGCTTG	
Igf2-WT-F	GGTGCCTCCTGTCTGGTAAC	
Igf2-WT-R	GCCACTCTATCTTCCTCGCC	
Zfp57-KO-F	CCTCAGGAAAGCTCTTGGCA	Genotyping of *Zfp57* knockout
Zfp57-KO-R	GGTGGTCTACCAGACACAAACA	
Zfp57-WT-F	GAGTCACAAAGTTCCGGGGT	
Zfp57-WT-R	GGCCTCCATGTGAACACCTA	
Tet1-sg	ATGATCACACTCCCCCCCGGAGG	sgRNA sequence
Tet2-sg	AAAGTGCCAACAGATATCCAGG	
Tet3-sg	AAGGAGGGGAAGAGTTCTCGAGG	
Dusp9-sg	GTCTGAGTCGGTCATGCCTGTGG	
H19-DMR-sg1	CATGAACTCAGAAGAGACTGAGG	
H19-DMR-sg2	AGGTGAGAACCACTGCTGAGTGG	
IG-DMR-sg1	CGTACAGAGCTCCATGGCACAGG	
IG-DMR-sg2	CTGCTTAGAGGTACTACGCTAGG	
Zfp57-sg1	ACCAGTCAGTTATGAGGACGTGG	
Zfp57-sg2	AAGTCCTGAATGCGTTGCCAAGG	
Zfp57-sg3	GATAGCCGAGCAAATGACCCAGG	
Zfp57-sg4	TAAGGGACTCCTCGGGAAAGAGG	
Dnmt1-sg1	TCGGAAGGATTCCACCAAGCAGG	
Dnmt1-sg2	ACAGCCGGAAAACACATCCAGGG	
Dnmt1-sg3	CGTGTCCTACAGACGCTCCATGG	
Dnmt1-sg4	ACTGTGACTACTACCGGCCTCGG	
Dnmt3a-sg1	ACATGCCTCCAATGAAGAGTGGG	
Dnmt3a-sg2	AATGAAGAGTGGGTGCTCCAGGG	

Dnmt3a-sg3	GCGGGCATAAGGGCACCTATGGG	
Dnmt3a-sg4	CGCACATGTAGCAGTTCCAGGGG	
Dnmt3b-sg	AGAGTGGGGCCCGTTCGACTTGG	
H19-gene-sg1	TGTCGTCCATCTCCGTCTGAGGG	
H19-gene-sg2	CAATATAATGCGACTCATGGGGG	
H19-gene-sg3	TGGCGGCTGGTCGGATAAAGGGG	
H19-gene-sg4	TTACTTTTGGTTACAGGACGTGG	
Igf2-sg1	CAGTTTGTCTGTTCGGACCGCGG	
Igf2-sg2	CCAACATCGACTTCCCCACTGGG	
Igf2-sg3	GATCAGGGGACGATGACGTTTGG	
Igf2-sg4	TCTCCGAAGAGGCTCCCCCGTGG	

Table S4. List of all maternal and paternal DMRs used in this study.

Name	Genome	MethAllele	Chromosome	Start	End
ZAC1	mm9	m	chr10	12810276	12810604
ZAC1	mm9	m	chr10	12810950	12811333
GRB10	mm9	m	chr11	11925485	11926335
U2AF1-RS1	mm9	m	chr11	22871842	22872319
PEG13	mm9	m	chr15	72636765	72642079
IGF2R/AIR	mm9	m	chr17	12934163	12935573
IMPACT	mm9	m	chr18	13130706	13132250
MCTS2	mm9	m	chr2	152512491	152513011
NNAT	mm9	m	chr2	157385786	157387398
NESPAS	mm9	m	chr2	174121208	174126482
GNAS-EXON1A	mm9	m	chr2	174152431	174154508
PEG10	mm9	m	chr6	4697209	4697507
MEST	mm9	m	chr6	30686488	30689335
NAP1L5	mm9	m	chr6	58856690	58857056
ZIM2	mm9	m	chr7	6680287	6684827
SNURF/SNRPN	mm9	m	chr7	67149878	67150301
NDN	mm9	m	chr7	69493100	69493581
INPP5F_V2	mm9	m	chr7	13583178	13583215

				8	6
KCNQ1OT1	mm9	m	chr7	150481060	150481397
KCNQ1OT1	mm9	m	chr7	150481504	150481527
GTL2/DLK1	mm9	p	chr12	110761563	110768989
H19/IGF2	mm9	p	chr7	149766168	149768424
RASGRF1	mm9	p	chr9	89774406	89774691

Supplementary Figures

Fig. S1. Derivation of AG-haESCs by different culture media.

A. Diagram for the generation of AG-haESCs from sperm-cloned blastocysts in serum/LIF (S/L), S/L supplemented with 2i (2i/L), or S/L with a2i (a2i/L) media. Haploid and diploid cells were labeled in blue and yellow, respectively. Haploid cells were enriched by FACS. 2i-7, 2i-12, 2i-14, and 2i-R9 are haploid cell lines derived in 2i/L medium. a2i-6 and a2i-8 are haploid cell lines derived in a2i/L medium. No AG-haESCs were successfully derived in S/L medium. FCS, fetal calf serum; FCAS, fluorescence-activated cell sorting.
B. Summary of diploid and haploid ESCs derived from androgenetic blastocysts in S/L, 2i/L, and a2i/L media.
C. Colony morphology of AG-haESCs cultured in 2i/L and a2i/L media.
D. Immunofluorescent staining of OCT4 and SOX2 in AG-haESCs cultured in 2i/L and a2i/L media.
E. SC pups from ICAHCI using the 2i/L-cultured AG-haESCs. Cell line 2i-12 was used in the experiment. Pups obtained by C-section from a pseudopregnant mouse at E19.5 are shown. Asterisks label growth-retarded SC pups.
F. DNA methylations of *H19*-DMR and *IG*-DMR in 2i/L-cultured AG-haESCs (2i-12) with different passages were analyzed by bisulfite PCR. Cultured spermatogonial stem cells (SSCs) (Wang et al., 2021) with hypermethylated *H19* and *IG* DMRs are a positive control. Open and filled circles represent unmethylated and methylated CpG sites, respectively.
G. SC pups from ICAHCI using the AG-haESCs cultured in a2i/L medium. The cell line a2i-8 was used in the experiment. Pups obtained by C-section from a pseudopregnant mouse at E19.5 are shown. The asterisk labels a growth-retarded SC pup.
H. DNA methylations of *H19*-DMR and *IG*-DMR in a2i/L-cultured AG-haESCs (a2i-8) with different passages were analyzed by bisulfite PCR.

Fig. S2. Characterization of TSa2i/L-derived AG-haESCs and SC mice obtained by ICAHCI.

A. Immunofluorescent staining of OCT4 and SOX2 in TSa2i/L-derived AG-haESCs (TSa2i-14).
B. Histological images of teratoma sections from TSa2i/L AG-haESCs showing the formation of three germ layers.
C. DNA methylations of *Rasgrf1* DMRs in TSa2i/L AG-haESCs (TSa2i-14, TSa2i-C57, and TSa2i-F1) with different passages determined by bisulfite PCR analysis.
D. SC pups generated by ICAHCI of haploid cells from the TSa2i-14. Note that the recipient female delivered by itself.
E. The sequences of *Tet1*, *2*, and *3* in *Tet*-triple-knockout (TKO) AG-haESCs.
F. Immunoblot analysis of DUSP9 and HA in HA-tagged *Dusp9* haESCs (5' HA-120, 125, and 138). The asterisk labels a non-specific band of DUSP9 antibody. GAPDH is a loading control.
G. Growth curve of SC mice generated from TSa2i/L-derived AG-haESCs (TSa2i-14 and TSa2i-C57), 2i/L-derived AG-haESCs with *H19* and *IG* DMR deletions (2i-O48) (Zhong et al., 2015), and sperm.

H-K. Transcriptional analysis of imprinted genes (*H19*, *Igf2*, *Gtl2*, and *Dio3*) in different tissues of E18.5 fetuses (generated from TSa2i-14, 2i-O48, and sperm), including placenta, lung, liver, kidney, heart, and brain (n=3 samples for each group). The expression values were normalized to that of *Gapdh*. All error bars indicate the average mean ±SEM. *, P<0.05; **, P<0.01; ****, P<0.0001.

Fig. S3. DNA methylation profiling of TSa2i/L-derived AG-haESCs with early and late passages.

A. Boxplot showing the DNA methylation levels of different gene elements in 2i/L, a2i/L, and TSa2i/L-derived AG-haESCs.
B. Heatmap showing the DNA methylation levels of maternal DMRs in TSa2i/L AG-haESCs. Majority of maternal DMRs except *Peg13*-DMR were hypomethylated in TSa2i/L-treated AG-haESCs.
C. The average DNA methylation levels of two somatic DMRs (*Igf2* and *Gtl2*) in 2i/L (2i-R9, 2i-7, and 2i-14) and TSa2i/L (TSa2i-14 and TSa2i-C57) AG-haESCs detected by bisulfite PCR combined with high-throughput sequencing. *Gtl2* DMR is located at the promoter of *Gtl2* and *Igf2* DMR is located at the gene body of *Igf2*.

Fig. S4. H3K9me3 distributions on the genome of 2i/L and TSa2i/L AG-haESCs.

A. Bar plot showing H3K9me3 levels at maternal and paternal DMRs for two alleles during early embryonic development. RPKM, reads per kilobase of bin per million reads sequenced. The original H3K9me3 ChIP-seq is from the published data (Wang et al., 2018).
B. Snapshot of H3K9me3 signals at *IG*-DMR for two alleles at early embryonic stages (Wang et al., 2018).
C. The H3K9me3 peak distribution in different genomic regions for 2i/L and TSa2i/L-derived AG-haESCs.
D. The H3K9me3 peak distribution in different repeat elements for 2i/L andTSa2i/L-derived cells.
E. Heatmap of the H3K9me3 levels around paternal DMRs, maternal DMRs, and non-DMRs in different cell lines. The total H3K9me3 peaks were called using the H3K9me3 ChIP-seq data from all TSa2i/L and 2i/L cells. The peaks which are not overlapped with gamete DMRs were named non-DMRs.
F. Snapshot of H3K9me3 at *Rasgrf1*-DMR for TSa2i/L AG-haESCs (TSa2i-14 and TSa2i-C57) with different passages.
G. The average distribution of ZFP57 around DMRs and non-DMRs for 2i/L and TSa2i/L-derived AG-haESCs. The ZFP57 peaks were called using TSa2i-C57 ChIP-seq data. The peaks overlapped with gamete DMRs were named DMRs. Otherwise, the peaks were called non-DMRs.
H. Immunoblot analysis of ZFP57 in WT (TSa2i-C57) and *Zfp57* KO cells.
I. Boxplot showing the DNA methylation levels of the ZFP57 peaks overlapped with DMRs or non-DMRs in TSa2i/L AG-haESCs and the same cells with *Zfp57* KO (growing for 20 passages).

Fig. S5. Epigenetic changes in AG-haESCs transferred from 2i/L to SF/a2i/L.

A. Dot blot analysis of 5mC in 2i/L-cultured cells (2i-14 and 2i-7) and the same cells transferred to SF/a2i/L medium for 5 passages (5-p) or 10 passages (10-p).

B. Immunoblot analysis of DNMT1, DNMT3A, DNMT3B, DNMT3L, UHRF1, and ZFP57 in AG-haESCs cultured in 2i/L (2i-14, p48 and 2i-7, p40) or switched to SF/a2i/L for 10 passages (2i-14-2i/L-SF/a2i-10-p and 2i-7-2i/L-SF/a2i-10-p). ACTB is a loading control.

C. Heatmap showing the increased H3K9me3 signals and DNA methylation levels in cells transferred from 2i/L to SF/a2i/L.

Fig. S6. Methylation heterogeneity of paternal DMRs in TSa2i/L-derived AG-haESCs.

A. SC pups from ICAHCI using TS/a2i-C57 AG-haESCs. Pups obtained by C-section from a pseudopregnant mouse at E18.5 are shown. The asterisk labels a growth-retarded SC pup.
B. Methylation state of the *H19*-DMR and *IG*-DMR in a normal SC pup and a retarded pup derived from TSa2i-C57 AG-haESCs.
C. Strategy of single-cell expansions of TSa2i-C57 AG-haESCs. The subclones (sc1 to sc21, see also Fig. 6C) obtained through the first round of single-cell expansion were divided into three groups according to DNA methylation levels of *H19*-DMR, i.e., highly, moderately, and lowly methylated cells. In the second round of single-cell expansion, subclones with high (sc6), medium (sc13), and low (sc16) levels of *H19*-DMR methylation were used. The DNA methylation levels of *H19* and *IG* DMRs for all subclones were analyzed.
D. The DNA methylation levels of *H19*-DMR and *IG*-DMR in TSa2i-14 AG-haESCs and the same cells with *Dnmt3a*/*3b* double knockout cultured for 10 passages or *Dnmt1* knockout cultured for 7 passages.
E. DNA methylation levels of *H19* and *IG* DMRs in subclones with different *H19*-DMR methylations (sc6, sc8, sc13, and sc16) before and after proliferation for 10 passages.
F. ChIP-qPCR analysis of H3K9me3 and H3K4me3 enrichment in different regions of subclones (sc6, sc13, and sc16). qPCR was performed with primers for paternal DMRs (*H19*, *IG*, and *Rasgrf1*-DMRs); the *Olfr9* gene was used as a negative control for H3K9me3 and H3K4me3 and promoter of *Actb* was used as a positive control for H3K4me3. All error bars indicate the average mean ±SEM. n=3 samples in each group. *, $P<0.05$; **, $P<0.01$; ****, $P<0.0001$.
G. Growth curve of selected subclones (sc6, sc8, sc13, and sc16) after growing for 9 or 14 passages (9-p or 14-p). All error bars indicate the average mean ±SEM. n=3 samples in each group.
H. Genotyping analysis of *Igf2* deletion in sc6 cells with *Igf2* knockout.
I. Growth curve of sc6-originated cells with hypermethylated *H19*-DMR and the same cells with *Igf2* knockout (*Igf2* KO-17, 63, 80, and 88 representing 4 different KO lines) in 120 hours. All error bars indicate the average mean ±SEM. n=3 samples in each group.
J. Genotyping analysis of *H19* gene deletion in sc16 cells with *H19*-gene knockout.
K. The ratio of cells with one set of chromosomes (1c) in the AG-haESCs with hypomethylated (2i-14-SF/a2i, 2i-7-SF/a2i representing 2i/L-SF/a2i/L-switched cells which sustain globally hypermethylated DNA but were free DNA methylation at paternal DMRs) and with hypermethylated *H19* and *IG* DMRs (TS/a2i-F1 and TSa2i-14) during cell passaging. All error bars indicate the average mean ±SEM. n=3 samples in each group.

L. Genotyping analysis of *H19*-DMR deletion in TSa2i-C57 cells with *H19*-DMR deletion.
M. Genotyping analysis of *IG*-DMR deletion in TSa2i-C57 cells with *IG*-DMR deletion.

Fig. S7. TSa2i/L-derived AG-haESCs sustain paternal DMR methylation state in culture without feeder cells.

DNA methylation levels of paternal DMRs in TSa2i/L-derived AG-haESCs cultured on feeders (mouse embryonic fibroblasts, MEF) for 10 passages or transferred from MEFs to Poly-Laminin, Matrigel, or Fibronectin and cultured for 10 passages. Imprints can be maintained without feeders at least in short-term culturing.

Figure S1

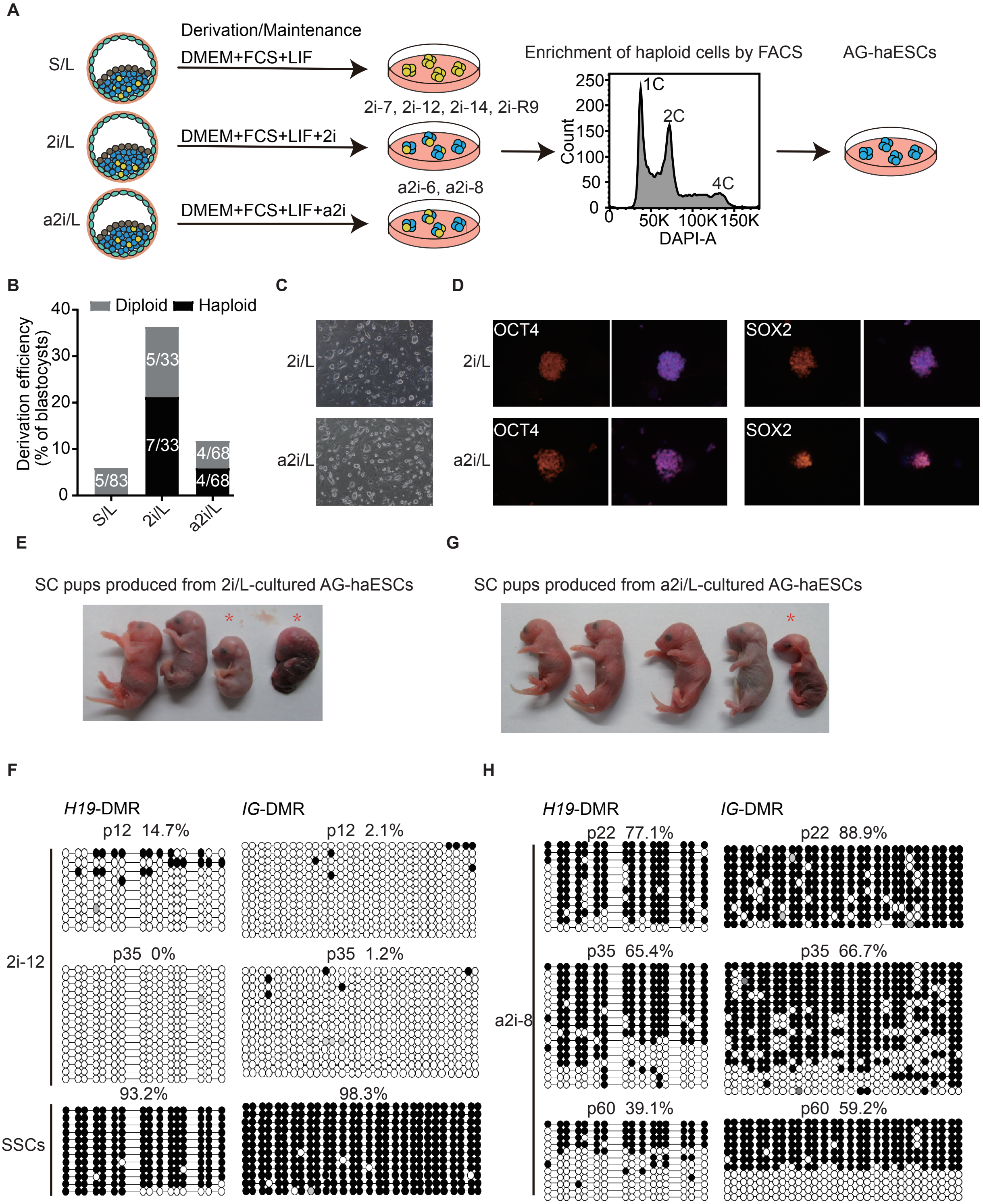
A
Derivation/Maintenance
S/L
DMEM+FCS+LIF
2i/L
DMEM+FCS+LIF+2i
a2i/L
DMEM+FCS+LIF+a2i
2i-7, 2i-12, 2i-14, 2i-R9
a2i-6, a2i-8
Enrichment of haploid cells by FACS
AG-haESCs
1C
2C
4C
Count
DAPI-A
B
Diploid
Haploid
Derivation efficiency (% of blastocysts)
5/83
5/33
7/33
4/68
4/68
S/L
2i/L
a2i/L
C
D
OCT4
SOX2
E
SC pups produced from 2i/L-cultured AG-haESCs
G
SC pups produced from a2i/L-cultured AG-haESCs
F
H19-DMR
IG-DMR
p12 14.7%
p12 2.1%
p35 0%
p35 1.2%
2i-12
93.2%
98.3%
SSCs
H
p22 77.1%
p22 88.9%
p35 65.4%
p35 66.7%
a2i-8
p60 39.1%
p60 59.2%

Figure S2

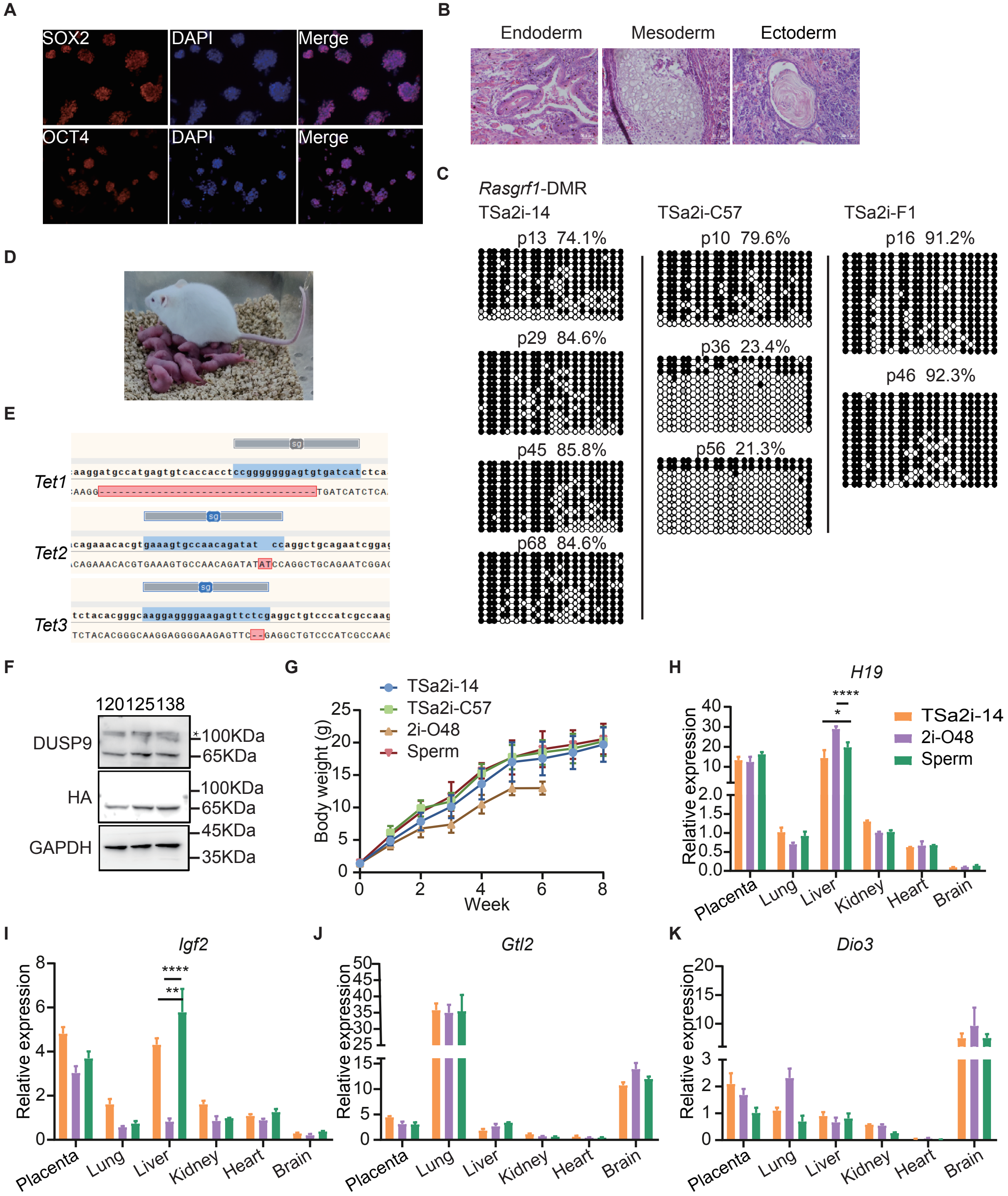
A
SOX2
DAPI
Merge
OCT4
DAPI
Merge
B
Endoderm
Mesoderm
Ectoderm
C
Rasgrf1-DMR
TSa2i-14
p13 74.1%
p29 84.6%
p45 85.8%
p68 84.6%
TSa2i-C57
p10 79.6%
p36 23.4%
p56 21.3%
TSa2i-F1
p16 91.2%
p46 92.3%
D
E
Tet1
Tet2
Tet3
sg
F
120125138
DUSP9
HA
GAPDH
100KDa
65KDa
100KDa
65KDa
45KDa
35KDa
G
TSa2i-14
TSa2i-C57
2i-O48
Sperm
Body weight (g)
Week
H
H19
TSa2i-14
2i-O48
Sperm
Relative expression
Placenta
Lung
Liver
Kidney
Heart
Brain
I
Igf2
Relative expression
J
Gtl2
Relative expression
K
Dio3
Relative expression

Figure S3

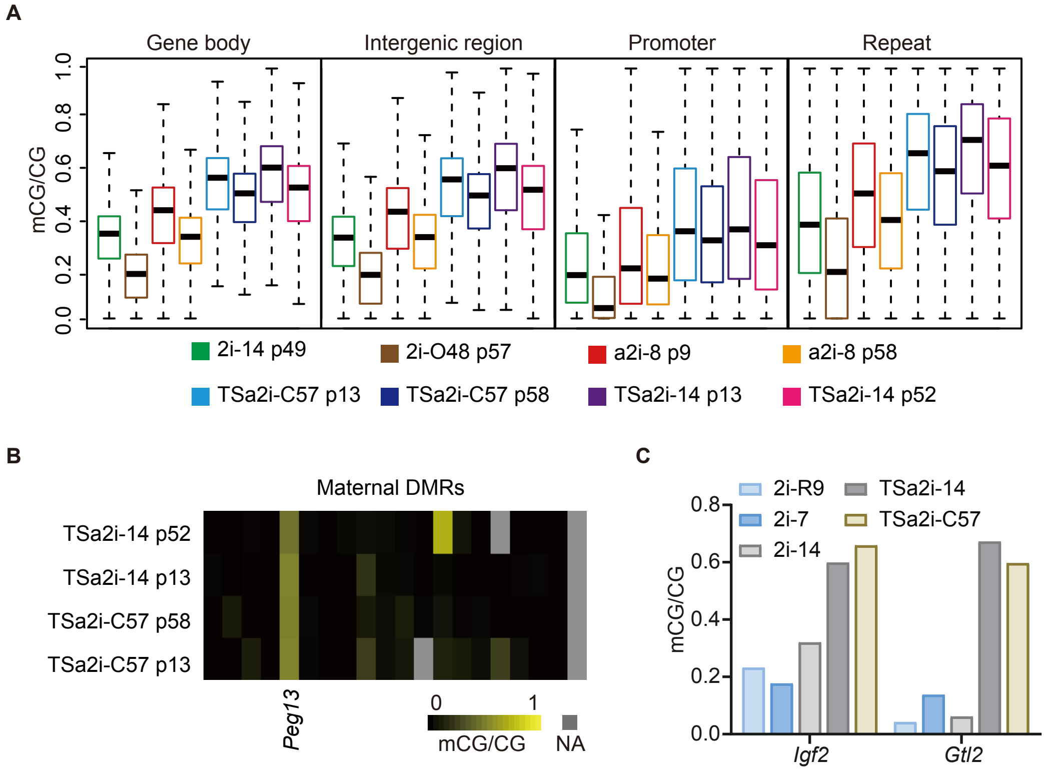
A
Gene body
Intergenic region
Promoter
Repeat
mCG/CG
0.0
0.2
0.4
0.6
0.8
1.0
2i-14 p49
2i-O48 p57
a2i-8 p9
a2i-8 p58
TSa2i-C57 p13
TSa2i-C57 p58
TSa2i-14 p13
TSa2i-14 p52
B
Maternal DMRs
TSa2i-14 p52
TSa2i-14 p13
TSa2i-C57 p58
TSa2i-C57 p13
Peg13
0
1
mCG/CG
NA
C
2i-R9
2i-7
2i-14
TSa2i-14
TSa2i-C57
mCG/CG
0.0
0.2
0.4
0.6
0.8
Igf2
Gtl2

Figure S4

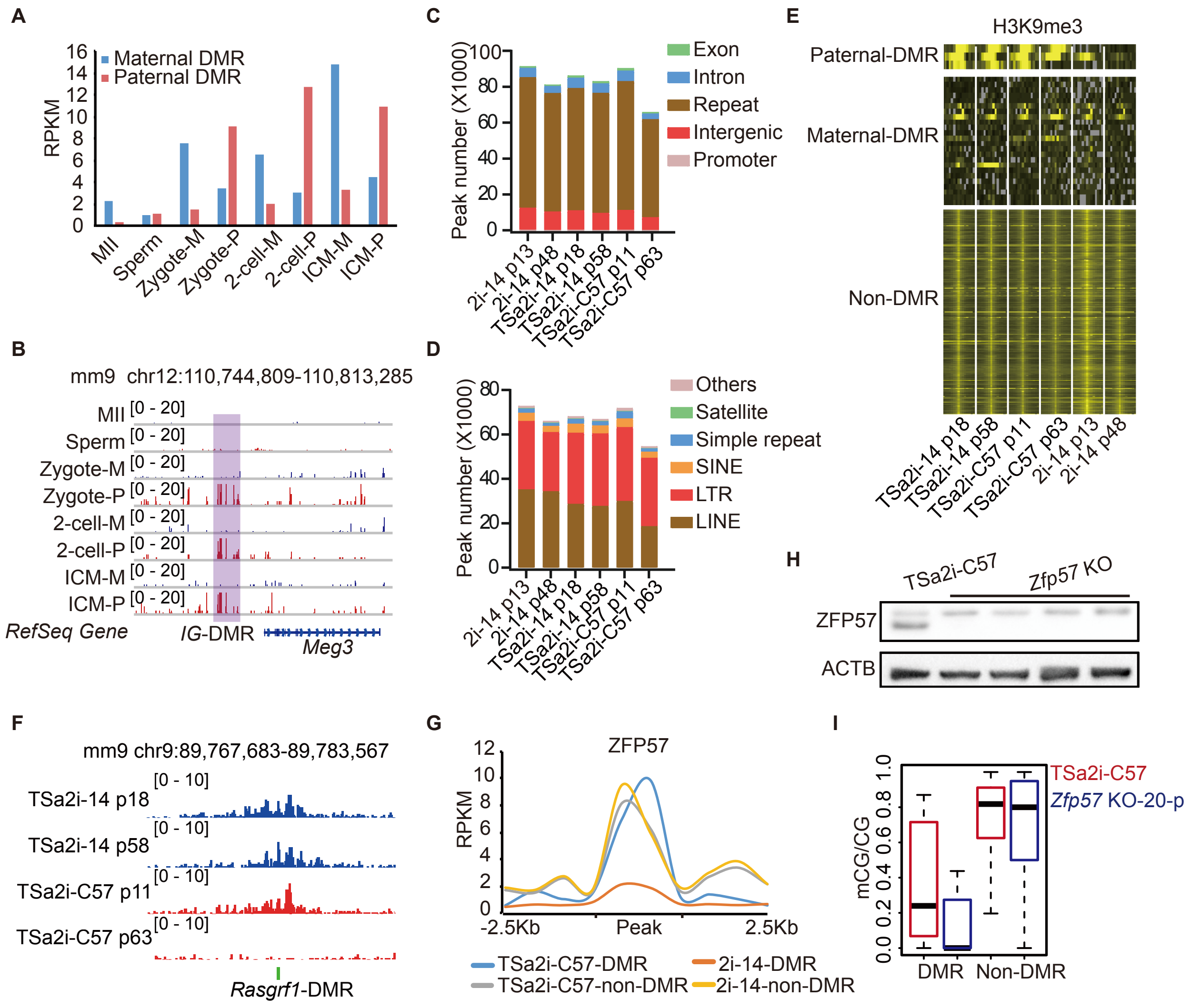
A
Maternal DMR
Paternal DMR
RPKM
MII
Sperm
Zygote-M
Zygote-P
2-cell-M
2-cell-P
ICM-M
ICM-P
B
mm9 chr12:110,744,809-110,813,285
MII [0 - 20]
Sperm [0 - 20]
Zygote-M [0 - 20]
Zygote-P [0 - 20]
2-cell-M [0 - 20]
2-cell-P [0 - 20]
ICM-M [0 - 20]
ICM-P [0 - 20]
RefSeq Gene
IG-DMR
Meg3
C
Peak number (X1000)
Exon
Intron
Repeat
Intergenic
Promoter
2i-14 p13
2i-14 p48
TSa2i-14 p18
TSa2i-14 p58
TSa2i-C57 p11
TSa2i-C57 p63
D
Peak number (X1000)
Others
Satellite
Simple repeat
SINE
LTR
LINE
E
H3K9me3
Paternal-DMR
Maternal-DMR
Non-DMR
TSa2i-14 p18
TSa2i-14 p58
TSa2i-C57 p11
TSa2i-C57 p63
2i-14 p13
2i-14 p48
H
TSa2i-C57
Zfp57 KO
ZFP57
ACTB
F
mm9 chr9:89,767,683-89,783,567
TSa2i-14 p18 [0 - 10]
TSa2i-14 p58 [0 - 10]
TSa2i-C57 p11 [0 - 10]
TSa2i-C57 p63 [0 - 10]
Rasgrf1-DMR
G
ZFP57
RPKM
-2.5Kb
Peak
2.5Kb
TSa2i-C57-DMR
2i-14-DMR
TSa2i-C57-non-DMR
2i-14-non-DMR
I
mCG/CG
TSa2i-C57
Zfp57 KO-20-p
DMR
Non-DMR

Figure S5

A

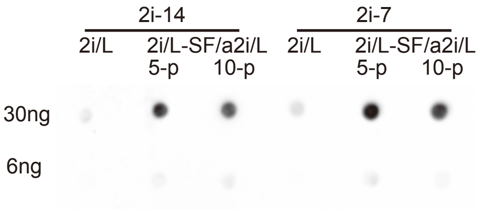

B

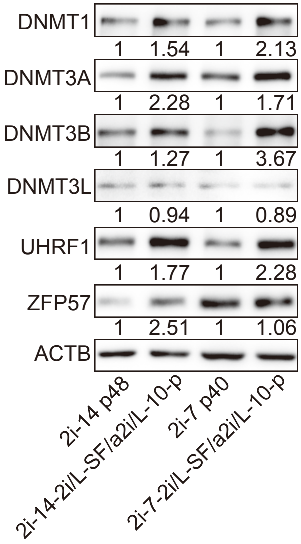

C

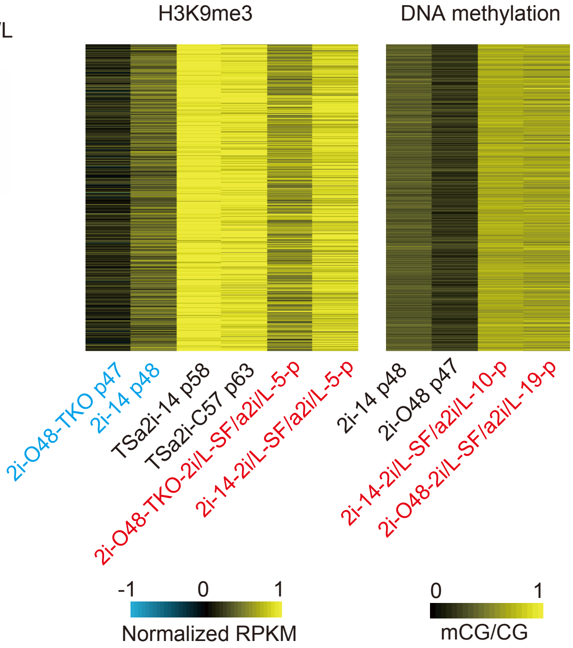

Figure S6

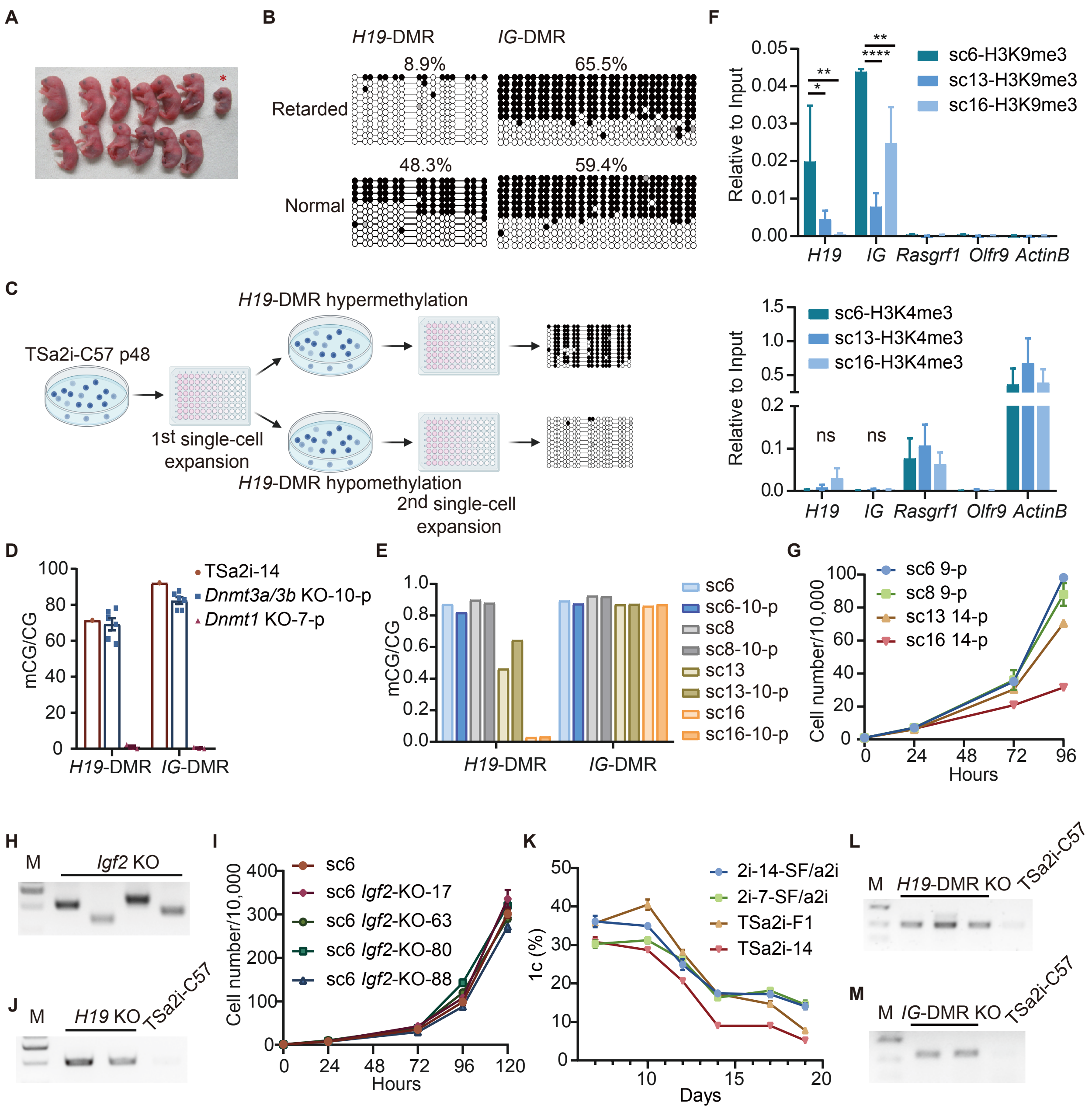

A
B
H19-DMR
IG-DMR
8.9%
65.5%
Retarded
48.3%
59.4%
Normal
F
0.05
0.04
0.03
0.02
0.01
0.00
Relative to Input
sc6-H3K9me3
sc13-H3K9me3
sc16-H3K9me3
H19
IG
Rasgrf1
Olfr9
ActinB
C
H19-DMR hypermethylation
TSa2i-C57 p48
1st single-cell expansion
H19-DMR hypomethylation
2nd single-cell expansion
sc6-H3K4me3
sc13-H3K4me3
sc16-H3K4me3
ns
ns
D
mCG/CG
TSa2i-14
Dnmt3a/3b KO-10-p
Dnmt1 KO-7-p
H19-DMR
IG-DMR
E
sc6
sc6-10-p
sc8
sc8-10-p
sc13
sc13-10-p
sc16
sc16-10-p
G
Cell number/10,000
sc6 9-p
sc8 9-p
sc13 14-p
sc16 14-p
Hours
H
M
Igf2 KO
I
sc6
sc6 Igf2-KO-17
sc6 Igf2-KO-63
sc6 Igf2-KO-80
sc6 Igf2-KO-88
K
1c (%)
2i-14-SF/a2i
2i-7-SF/a2i
TSa2i-F1
TSa2i-14
Days
L
H19-DMR KO
TSa2i-C57
J
H19 KO
M
IG-DMR KO

Figure S7

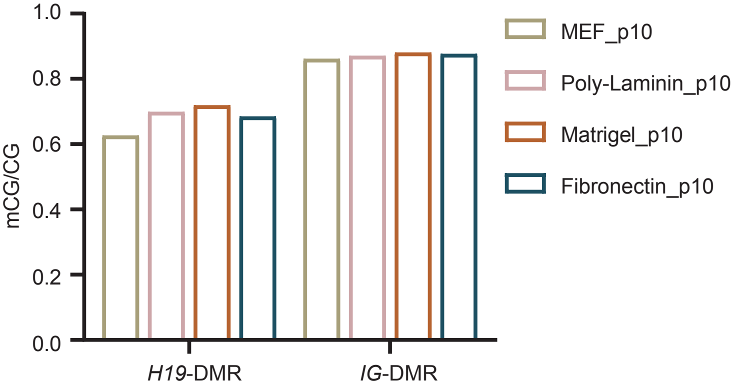

1.0
0.8
0.6
0.4
0.2
0.0
mCG/CG
H19-DMR
IG-DMR
MEF_p10
Poly-Laminin_p10
Matrigel_p10
Fibronectin_p10
